# Multisensory Interactions in Head and Body Centered Perception of Verticality

**DOI:** 10.3389/fnins.2020.599226

**Published:** 2021-01-12

**Authors:** Ksander N. De Winkel, Ellen Edel, Riender Happee, Heinrich H. Bülthoff

**Affiliations:** ^1^Intelligent Vehicles Research Group, Faculty 3mE, Cognitive Robotics Department, Delft University of Technology, Delft, Netherlands; ^2^Department of Perception, Cognition and Action, Max Planck Institute for Biological Cybernetics, Tübingen, Germany

**Keywords:** somatosensation, proprioception, vision, vertical, rod and frame, postural vertical, multisensory perception and integration

## Abstract

Percepts of verticality are thought to be constructed as a weighted average of multisensory inputs, but the observed weights differ considerably between studies. In the present study, we evaluate whether this can be explained by differences in how visual, somatosensory and proprioceptive cues contribute to representations of the Head In Space (HIS) and Body In Space (BIS). Participants (10) were standing on a force plate on top of a motion platform while wearing a visualization device that allowed us to artificially tilt their visual surroundings. They were presented with (in)congruent combinations of visual, platform, and head tilt, and performed Rod & Frame Test (RFT) and Subjective Postural Vertical (SPV) tasks. We also recorded postural responses to evaluate the relation between perception and balance. The perception data shows that body tilt, head tilt, and visual tilt affect the HIS and BIS in both experimental tasks. For the RFT task, visual tilt induced considerable biases (≈ 10° for 36° visual tilt) in the direction of the vertical expressed in the visual scene; for the SPV task, participants also adjusted platform tilt to correct for illusory body tilt induced by the visual stimuli, but effects were much smaller (≈ 0.25°). Likewise, postural data from the SPV task indicate participants slightly shifted their weight to counteract visual tilt (0.3° for 36° visual tilt). The data reveal a striking dissociation of visual effects between the two tasks. We find that the data can be explained well using a model where percepts of the HIS and BIS are constructed from *direct* signals from head and body sensors, respectively, and *indirect* signals based on body and head signals but corrected for perceived neck tilt. These findings show that perception of the HIS and BIS derive from the same sensory signals, but see profoundly different weighting factors. We conclude that observations of different weightings between studies likely result from querying of distinct latent constructs referenced to the body or head in space.

## Introduction

We are generally well aware of the orientation of our head and body with respect to gravity. Percepts of verticality are essential in all conditions where we have to stabilize ourselves, such as while standing or walking. Ample studies have shown that postural control (Van der Kooij et al., 1999, 2001; Oie et al., 2002; Peterka, 2002; Carver et al., 2005; Happee et al., 2017) and perception of verticality (Eggert, 1998; Barnett-Cowan et al., 2005, 2011, 2013; Dyde et al., 2006; Barnett-Cowan and Harris, 2008; Vingerhoets et al., 2009; Clemens et al., 2011; De Winkel et al., 2012, 2018b; Alberts et al., 2016) derive from integration of sensory signals from the visual system and sensory organs responsive to gravito-inertial stimulation, and prior knowledge that “up” is usually above the head. It is generally accepted that the central nervous system constructs these percepts in a fashion that resembles calculating a vector sum (Mittelstaedt, 1983; Oman, 2003). More recently, this notion has been reinterpreted as a reflection of the nervous system performing statistical inference (Eggert, 1998; Dyde et al., 2006; Clemens et al., 2011; De Winkel et al., 2018b). According to these statistical models, a percept is a value for the inferred variable that maximizes the likelihood of sensory signals given the variable [i.e., Maximum Likelihood Estimation (MLE), e.g., Ernst and Banks, 2002; Hillis et al., 2002; Ernst and Bülthoff, 2004], or a value that maximizes the posterior probability of the variable given the sensory signals; after factoring in prior knowledge [i.e., the Maximum A-Posteriori (MAP) estimate, e.g., Yuille and Bülthoff, 1996]. Provided that certain assumptions are met, these models essentially predict that percepts are weighted averages of the constituent signals, with weights that are inversely proportional to each signal's variance (see Li et al., 2020 for a concise overview).

Experimental studies where the perception of verticality was modeled in this way however vary considerably with respect to the observed weights for different signals. For instance, Dyde et al. (2006) found that when participants reported a subjective visual vertical by aligning an object in the visual display with the perceived gravitational vertical, inertial (vestibular, somatosensory) signals were most important, followed by prior knowledge, and finally visual signals. In contrast, when participants reported the perceived upright indirectly, by their interpretation of the ambiguous symbol “p” (or “d”) relative to their orientation, prior knowledge was most important, followed by visual signals, and with inertial signals being of least importance. Such differences in relative weights are not readily explained by a vector sum model or by its recent statistical reinterpretations (Dyde et al., 2006; De Winkel et al., 2018a): even though the specifics of different experimental setups may affect the variance of sensory signals, it is not clear why simply performing different tasks in near identical conditions would affect the variance of sensory signals themselves.

An explanation for the different weightings observed between studies is that different tasks probe different internal representations of verticality (Dyde et al., 2006; Angelaki and Cullen, 2008; Clemens et al., 2011; Fraser et al., 2015). It is often implicitly assumed that *the* internal representation of verticality is head-centered. Introspectively, we can however differentiate between representations of verticality corresponding to the orientation of different parts of the body relative to gravity. This notion is supported by studies that distinguish between perception of the HIS and the BIS, relative to gravity (Mittelstaedt, 1995, 1998; Clemens et al., 2011). The existence of parallel representations could also explain why stroke patients with vestibular lesions can present two distinct perceptual distortions: there are those who perceive their visual environment to be tilted, but who have relatively unimpaired representations of body tilt relative to gravity, and those who have a distorted perception of body tilt, but who have a relatively unimpaired representation of the orientation of the visual environment (Bisdorff et al., 1996; Anastasopoulos et al., 1997; Karnath et al., 2000, 2005).

Consequently, the finding of different weightings between studies that employ different experimental tasks may reflect that responses on these tasks result from queries of different latent constructs. Clemens et al. (2011) investigated how different representations of verticality could be constructed from signals provided by different sensors. Specifically, they aimed to delineate interactions between the vestibular system in the inner ear, somatic graviceptors in the trunk (Mittelstaedt, 1996; Vaitl et al., 2002), and neck proprioceptors. They proposed a Bayesian model where estimates of the HIS and BIS are constructed as combinations of (1) sensory signals from the vestibular and somatosensory systems that inform of these constructs directly; (2) prior knowledge, namely that the gravitational vertical usually aligns with the long body axis, and that the head is aligned with the long body axis (Mittelstaedt, 1983); and (3) indirect estimates of the HIS and BIS, that correspond to the alternative representation of verticality corrected for tilt of the head relative to the body [i.e., BIS+Head On Body (HOB) for the HIS, and HIS-HOB for BIS], using information provided by neck proprioceptors (Clemens et al., 2011; Kheradmand and Winnick, 2017; Medendorp et al., 2018). In an experiment, the authors seated participants in a tilting chair in complete darkness, and probed their representations of the HIS and BIS using two tasks: the HIS was probed using a subjective visual vertical task, where participants were tilted physically to predefined target angles and then provided judgments on whether the orientation of a visually presented luminous line was either clockwise or counter clockwise relative to gravity; and the BIS was probed using a subjective body tilt task, where participants judged whether body tilt stimuli were either clockwise or counter clockwise relative to predefined target angles. The proposed model could account for the data well, and performed better than an alternative model that included only direct pathways, thus supporting the notion that there are interactions between different internal representations of verticality when vision is excluded.

In the present study, we investigate conditions with vision to determine whether the reported differences in sensory weightings can be explained by differences in internal representations of the HIS and BIS. We expand upon the work by Clemens et al. (2011) by incorporating vision in an adaptation of their model. Moreover, we evaluate whether perceptual effects generalize to standing posture maintenance. Participants were placed on a force plate that was mounted to a motion platform while wearing an “‘Alternative Reality' (AR)” head-mounted display system. This system showed them a live, stereoscopic video feed of their actual surroundings, at any desired tilt angle (De Winkel et al., 2018a; Nestmann et al., 2020). Using this setup, participants were presented with various (in)congruent combinations of physical and visual head and body tilt stimuli. Participants performed two tasks, believed to probe the HIS and BIS, respectively: in the RFT task, participants aligned a virtual visual rod with their perception of verticality; in the SPV task, participants adjusted the orientation of the motion platform such that it was perceived as upright. We chose to study verticality perception in standing participants because perception of the HIS and BIS are of particular relevance to posture maintenance, and because previous work (Nestmann et al., 2020) suggested that the AR setup may have applied therapeutic value in certain neurological disorders characterized by misperceptions of verticality that cause patients to fall (e.g., Pusher syndrome Karnath et al., 2000, 2005). We first assessed the contributions of different sensory signals with Linear Mixed Effects (LME) models, and subsequently evaluated whether our verticality perception model can provide a parsimonious account of the experimental data.

## Methods

###  Participants

Ten people took part in this study. The sample was made up of Max Planck Institute employees and people recruited from the institute participant database (mean age: 31.4 years, sd: 13.6, 6 female). External participants were compensated for their time at a rate of € 8/h.

###  Setup

**Figure 1:**
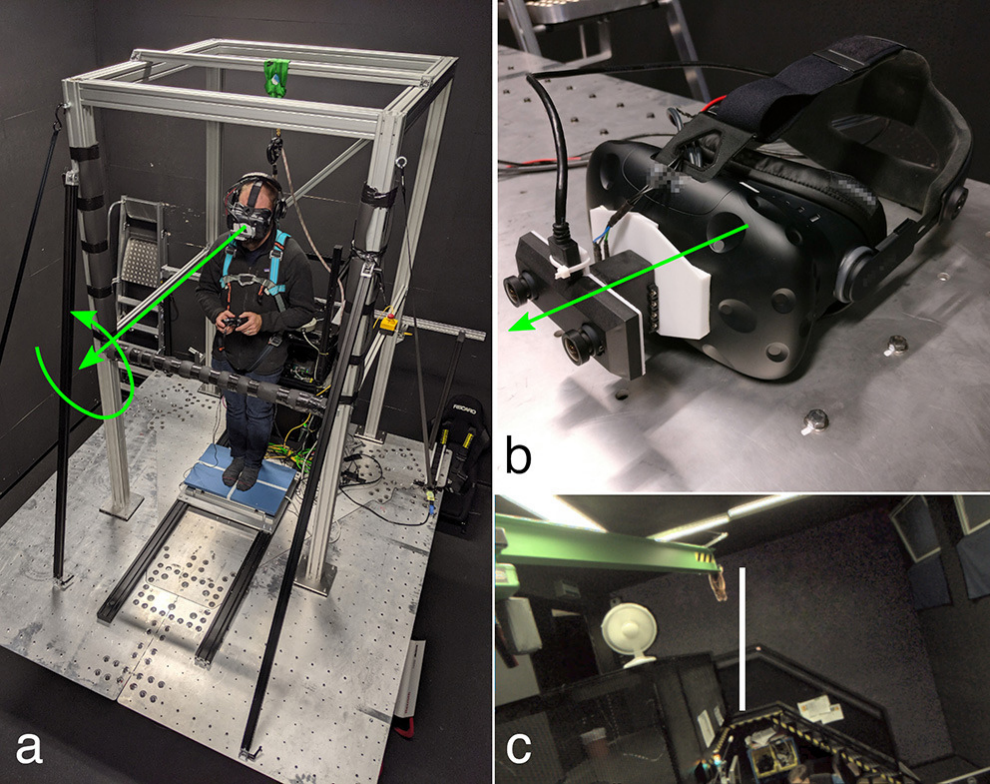
Photographs of the apparatus. The left panel **(a)** shows a participant standing on the force plate on top of the motion platform. The participant is loosely secured by the harness to the top of a frame mounted to the motion platform. He is holding a game controller, and is wearing the AR system, shown in detail in the top right panel **(b)**. The green arrows represent the axes of rotation of the platform **(a)** and the AR system **(b)**. These coincide with the participant's naso-occipital axis. The bottom right panel **(c)** shows a (monocular) screenshot of the participant's view of the room (showing i.e., stairs, green crane, white fan), including the rod used in the RFT task.

The experimental setup consisted of a motion platform, the AR system, and a force plate. A birds-eye view of the setup is shown in the left panel of Figure 1.

The motion platform is an eMotion 1500 hexapod motion system (Bosch Rexroth AG, Lohr am Main, Germany), which features six actuated legs that allow motion of the platform in six degrees of freedom. In the present experiment, only roll motion was presented.

The AR system consists of an OVRVision Pro stereo camera (Wizapply, Osaka, Japan) mounted via a Dynamixel AX12-A servo motor (Robotis, Lake Forest, California, United States) to a Vive head-mounted display (HTC, New Taipei City, Taiwan). The images captured by the cameras are displayed in the respective screens with an update rate of 45 frames per second. The resolution of the screens is 1, 080 × 1, 200 px, with a field of view of 100 × 110°. This corresponds to approximately 11 px per degree. The servo motor allows for rotation with an accuracy of 0.29°. The AR system is shown in the top right panel of Figure 1. A monocular screenshot of the view inside the head-mounted display for one of the experimental tasks is shown in the bottom right panel of Figure 1.

The force plate mounted to the platform is an AMTI BO400600-OP-2K-STT (Advanced Mechanical Technology, Inc., Watertown, Massachusetts, United States). It measures the forces and moments applied to its top surface. From these moments and forces, we calculated the Center Of Pressure (COP) of participants' bodies. The force plate is the blue plate on which the participant is standing in the left panel of Figure 1. Samples were collected at a rate of 250 Hz.

Participants were loosely secured to the platform using a climbing harness that was suspended from the top of the frame surrounding them. Actuator noise was masked by having participants wear a wireless headset (Plantronics, Santa Cruz, California, United States) that provided active noise cancellation. Participants provide inputs to the system with a hand held XBox game controller (Microsoft, Redmond, Washington, United States).

All the systems were controlled via a central real-time computer (Speedgoat GmbH, Liebefeld, Switzerland) that ran a Simulink model (The MathWorks, Inc., Natick, Massachusetts, United States).

### Conventions and Definitions

The motion platform and force plate use a right-handed rectangular coordinate system. The X, Y, and Z axes correspond to the surge/roll(φ) axis, the sway/pitch(θ) axis, and the heave/yaw(ψ) axis, respectively. Taking on the perspective of a participant, who is standing on top of the force plate on the motion platform, positive values correspond to forward translation/clockwise rotation (X-axis), rightward translation/nose-up rotation (Y-axis), and downward translation/rightward rotation (Z-axis). The platform motion reference point was offset for each participant, such that the X-axis aligned with the individual's naso-occipital axis.

**Figure 2:**
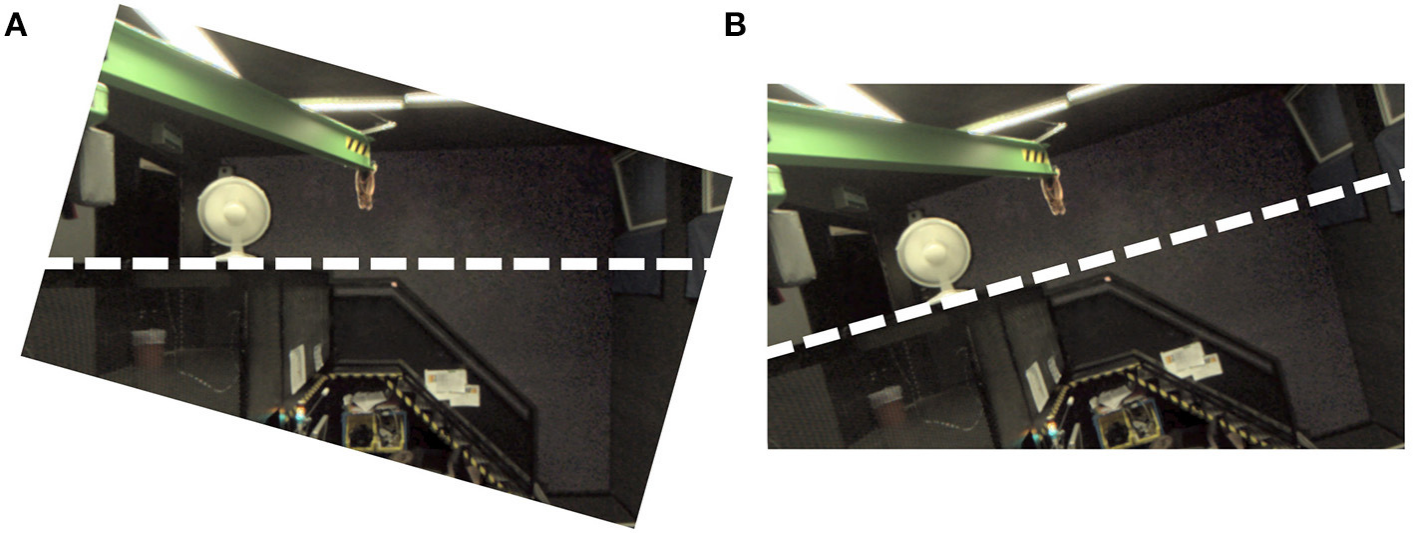
Demonstration how visual tilt stimuli were generated using the AR setup. Both panels show a monocular screenshot from the AR system. **(A)** Shows the result of tilting the cameras on the AR setup; **(B)** Shows the view after the captured image is transferred to the head-mounted display coordinate system. Note how clockwise camera tilt results in a visual stimulus that shows a counter-clockwise tilted environment. The thick dashed lines are added to emphasize the horizon. They were not shown in the actual experiment.

The AR system only allows roll rotations (i.e., about the X-axis), where the camera rotation axis was aligned with the naso-occipital axis. From the perspective of a participant, positive angles correspond to clockwise camera tilt. Visual tilt stimuli are created by projecting the image captured by the camera onto a fronto-parallel virtual surface that is aligned with the reference frame of the head-mounted display. Taken together, *clockwise* camera tilt results in *counter-clockwise* visual tilt stimuli (see Figure 2), which are indicative of *clockwise* head tilt.

Stimulus roll-tilt angles are denoted φ_*m*_, where the subscript *m* designates the manipulated modality, being either P for platform tilt; N for neck tilt; AR for AR system camera tilt, or V for visual tilt. Platform, neck and AR system tilt are manipulated experimentally. Visual tilt is a function of the experimentally manipulated tilt stimuli:

(1) $$\begin{array}{l} \varphi_{\text{V}}=\varphi_{\text{P}}+\varphi_{\text{N}}+\varphi_{\text{AR}}. \end{array}$$

In reality, perceived tilts depend on body tilt (φ_B_), which may differ from platform tilt because participants were standing semi-freely. However, an evaluation of perception model fits did not yield different conclusions depending on whether differences between platform and body tilt were included (see section 3.2). For simplicity, we therefore treated body tilt as if it was equal to platform tilt.

The physical stimuli are unknown to the nervous system; it can only make inferences based on signals provided by the sensory systems. Roll tilt signals from the sensory systems are denoted with letters *x*_*n*_, where the subscript *n* designates the sensory modality, being either *vis* for the visual system, *ves* for the vestibular system, *pro* for proprioceptors in the neck, and *som* for the somatic graviceptors in the trunk. The visual and vestibular systems sense the orientation of the HIS; somatic graviceptors sense the orientation of the BIS; and neck proprioceptors sense the orientation of the HOB.

Responses on the experimental tasks will be denoted with the letter *r*. A complete list of symbols is included as an Appendix.

### Rod and Frame Test

In the classical RFT (Witkin and Asch, 1948) task, participants are shown a rod that is presented against the background of a (tilted) frame, and asked to align the rod with what they believe is upright. Many adaptations of this task have been used since (e.g., De Winkel et al., 2012, 2018b; Alberts et al., 2016). We adopted this task using the AR system. As a rod, a rectangle of 50 × 2 cm was added to the middle of the virtual scene, 1m in front of the participant (see Figure 1c). Participants could rotate the rod around its center point using the left and right bumper buttons on the XBox controller (see Figure 1). This rod was not shown in the SPV task (described below). It was explained to participants that visual and inertial cues could be manipulated independently, and that they should adjust the orientation of the rod such that it aligned with the direction of gravity. A copy of the instructions is included as Supplementary Material.

On an individual trial, the camera of the AR system was tilted to one of the following angles: $\varphi_{\text{AR}}={\left[-36,-13,-5-,0,5,13,36\right]}^{{}^{\circ}}$, and the platform itself was tilted to one of the following tilt angles: $\varphi_{\text{P}}={\left[-3,0,3\right]}^{{}^{\circ}}$. The task was performed twice, in two separate experimental blocks: once with the head upright, and once with the head tilted to the right (clockwise; leaning on a cervical collar ≈ 15°). In the block with the head upright, each combination of φ_AR_, φ_P_ was presented three times (four times for participants 1 and 2), for a total of 7(φ_AR_) × 3(φ_P_) × 3(repetitions) = 63 trials (84 for participants 1 and 2); in the block with the head tilted, each combination was presented twice (three times for participants 1 and 2), for a total of 7(φ_AR_) × 3(φ_P_) × 2(repetitions) = 42 trials (63 for participants 1 and 2). The number of repetitions was reduced in the latter block to keep discomfort to a minimum. The motion profile that was used to change camera and platform tilt between trials followed a single period of a raised cosine bell in velocity, with a duration of (3 + ε)s, where ε ∈ [0, 0.2, 0.4, 0.6, 0.8, 1.0], chosen at random. This was done to confuse the relation between duration of the inter-trial rotation and tilt angle.On each trial, the initial angle of the rod was chosen at random from a uniform distribution over the range ±45°. This range was limited to avoid confusion in the data analysis on which end of the rod was intended to point up.

The reference frame for responses on the RFT task is provided by the head-mounted display. This means that a response $r_{\text{RFT}}=0^{{}^{\circ}}$ reflects alignment of the rod with the head, regardless of the orientation of the head relative to gravity or any visual influences. In order to align the rod with the gravitational vertical, participants must correct the rod for perceived tilt of the HIS. We therefore assume that

(2) $$\begin{array}{l} r_{\text{RFT}}=-\hat{\text{HIS}}, \end{array}$$

where $\hat{\text{HIS}}$ is the perceived tilt of the head relative to gravity.

### Subjective Postural Vertical

For the SPV task (Nestmann et al., 2020), participants were standing on the motion platform while wearing the AR system, just as in the RFT task. Here, the AR system only showed a (tilted) feed-through version of their surroundings; the rod was not shown.

The experimental conditions were identical to those of the RFT task, that is, the AR angles were $\varphi_{\text{AR}}={\left[-36,-13,-5-,0,5,13,36\right]}^{{}^{\circ}}$; platform tilt angles were $\varphi_{\text{P}}={\left[-3,0,3\right]}^{{}^{\circ}}$; and the task was performed once with the head upright and once with the head tilted to the right, in two separate experimental blocks. In the block with the head upright, there were three repetitions of each condition (four for participants 1 and 2), for a total of 7(φ_AR_) × 3(φ_P_) × 3(repetitions) = 63 trials (84 for participants 1 and 2); in the block with the head tilted to the right there were two repetitions (three times for participants 1 and 2), for a total of 7(φ_AR_) × 3(φ_P_) × 2(repetitions) = 42 trials (63 for participants 1 and 2).

For each trial, after the trial's target visual and physical tilt angles were reached, the task participants had to perform was to adjust the orientation of the platform until they believed that it was upright again (i.e., the platform was perpendicular to gravity). Participants controlled the orientation of the platform using the left and right bumper buttons on the XBox controller (see Figure 1). An isolated button press incremented the tilt by ±0.05°, but the signal was passed through an integrator and subsequently through a low-pass filter such that sustained button presses resulted in a higher rate of change, and such that motion was smooth. The platform maximum tilt angle was limited to ±5°, to prevent participants from falling over. Camera tilt was adjusted for platform tilt in real time such that the visual tilt angle relative to gravity remained constant within an experimental trial. Thereby visual cues of verticality became insensitive to platform roll and did not support the SPV task. The temporal transition of tilt angles between trials was achieved in the same way as in the RFT task. The platform tilt angle serves only as an initial position on each trial, similar to the randomized initial setting of the rod for each trial in the RFT task. It is therefore not expected to affect the results.

The reference frame for responses on the SPV task is provided by the motion platform. This means that a response $r_{\text{SPV}}=0^{{}^{\circ}}$ reflects that the BIS is perceived as upright when it is objectively aligned with the gravitational vertical. We assume that participants attempt to adjust the platform orientation to achieve a percept of upright body orientation given neck and AR tilt stimuli. Consequently,

(3) $$\begin{array}{l} r_{\text{SPV}}=\varphi_{\text{P}}\text{ such that }f\left(\varphi_{\text{P}};\varphi_{\text{N}},\varphi_{\text{AR}}\right)=\hat{\text{BIS}}=0^{{}^{\circ}}. \end{array}$$

Here $\hat{\text{BIS}}$ is the perceived body tilt, and *f*(φ_P_; φ_N_, φ_AR_) is the function that describes how it is constructed. As an intuitive explanation, consider that participants can correct any illusory body tilt due to AR tilt by counter rotating the platform.

### Verticality Perception Model

**Figure 3:**
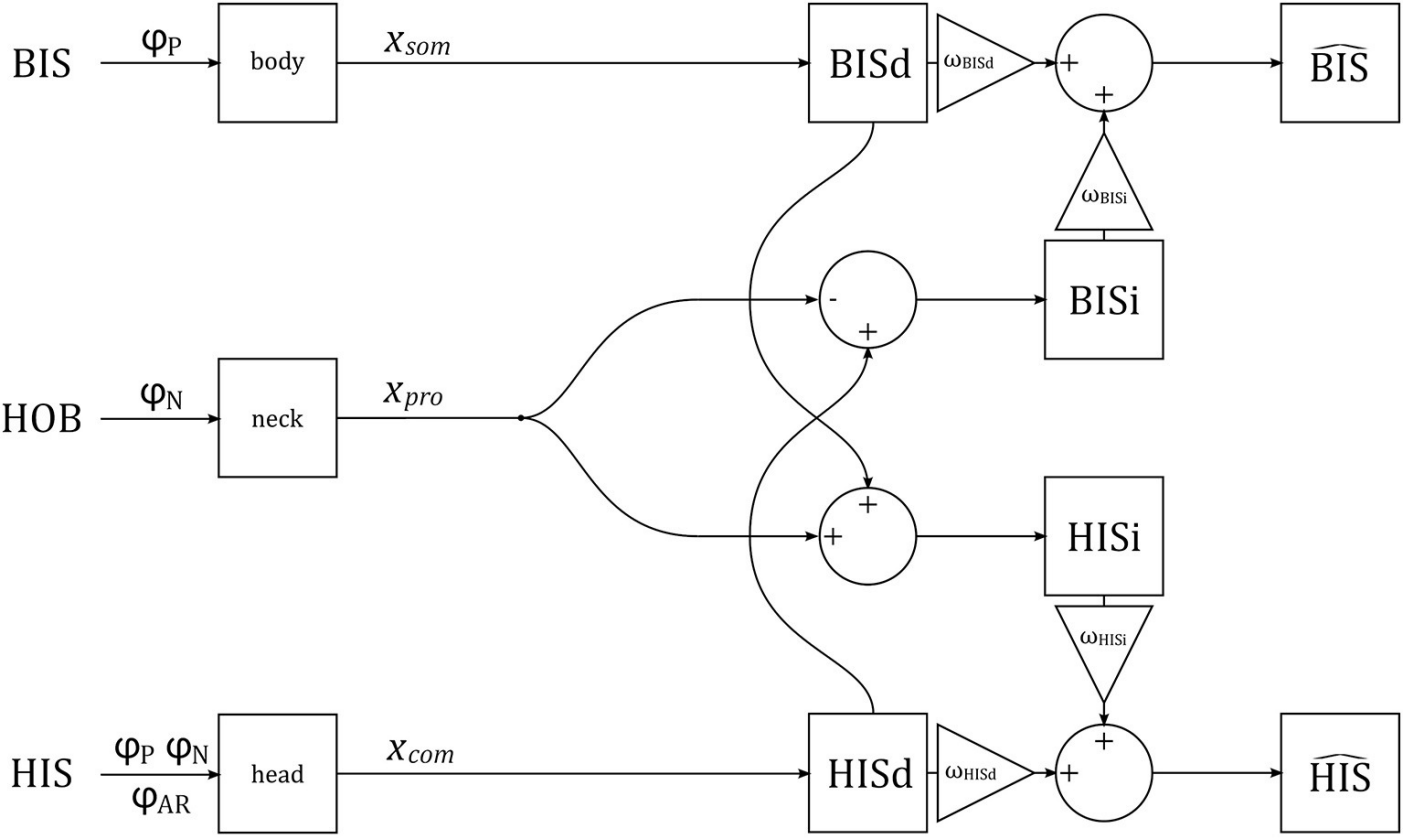
A schematic representation of the verticality perception model. The orientations of the BIS, HOB, and HIS are manipulated experimentally, via stimuli φ_P_, φ_N_, φ_AR_. The stimuli are transduced by somatosensory neurons in the body, proprioceptive sensors in the neck, and combined visual and vestibular sensors in the head, generating signals *x*_som_, *x*_pro_ and *x*_com_, respectively. These signals are used to generate direct (BISd, HISd) and indirect (BISi, HISi) representations of the BIS and HIS, which are then combined into estimates $\hat{\text{BIS}}$ and $\hat{\text{HIS}}$.

We assume that the brain constructs separate representations of head and body tilt using visual, vestibular, proprioceptive, and somatosensory signals. Some of these signals are directly representative of either head or body tilt, and can be used to generate *direct* estimates of head and body tilt, HISd and BISd. These direct estimates can also be corrected for neck tilt to generate alternative *indirect* estimates of tilt, HISi and BISi. The direct and indirect representations provide redundant information, and can be combined (with weighting factors ω, see below) to improve precision of the final estimates $\hat{\text{HIS}}$ and $\hat{\text{BIS}}$ (Clemens et al., 2011; Kheradmand and Winnick, 2017; Medendorp et al., 2018). These final estimates correspond to perception. A schematic representation of the verticality perception model is given in Figure 3.

We represent signals *x*_*n*_ from the sensory systems *n* = {*vis, ves, pro, som*} (see section 2.4) as unbiased normal distributed random variables with mean μ_*n*_ and standard deviation σ_*n*_:

(4) $$\begin{array}{l} x_{n}\sim N\left(\mu_{n},\sigma_{n}\right). \end{array}$$

Assuming that participants' bodies are rigid relative to the platform (see section 3.2 for evaluation of this assumption), the somatosensory signal on body tilt *x*_*som*_ has its mean equal to platform tilt, μ_*som*_ = φ_P_, which is known and manipulated experimentally, and standard deviation σ_*som*_, which was treated as a free parameter.

The proprioceptive signal on neck tilt *x*_*pro*_ has mean μ_*pro*_ = φ_N_, which was approximately 0° with the head upright, and around 15° with the head tilted, but was treated as a free parameter to account for unmeasured variation between individuals. Its standard deviation σ_*pro*_ was also treated as a free parameter.

Theoretically, the vestibular tilt signal has mean μ_*ves*_ = φ_P_ + φ_N_; and the mean of a visual tilt signal is determined by head tilt and tilt of the AR system, such that μ_*vis*_ = φ_P_ + φ_N_ + φ_AR_ (see also section 2.4). Following the MLE framework, head tilt could be estimated as a combination of μ_*ves*_ and μ_*vis*_, weighted by a function of the variances (Ernst and Banks, 2002; Hillis et al., 2002; Ernst and Bülthoff, 2004). However, including this combination in the modeling caused problems in uniquely estimating the model parameters (see section 4). We therefore treat the visual and vestibular systems as a combined head tilt sensor, producing a signal *x*_*com*_ with mean μ_*com*_ = φ_P_ + φ_N_ + φ_AR_ and standard deviation σ_*com*_. In line with the literature on visual perception, the standard deviation of the head tilt signal was allowed to increase with AR tilt angle (De Winkel et al., 2015, 2017, 2018b; Acerbi et al., 2018). This was modeled by allowing σ_*com*_ to increase with the eccentricity of φ_AR_: σ_*com*_ = *K*_*com*_|φ_AR_| + σ_*com*_0__. Here σ_*com*_0__ is the baseline noise level and *K*_*com*_ is a gain.

Next, we postulate that somatosensory signals *x*_*som*_, originating from a variety of sensors in the trunk and lower extremity (Mittelstaedt, 1996; Zaichik et al., 1999; Vaitl et al., 2002), yield a direct internal representation of the BIS, BISd; and signals by the head sensors *x*_com_ yield a direct internal representation of the HIS, HISd. Alternatively, somatosensory signals *x*_*som*_ can be used to estimate the BIS after correcting for tilt of the HOB, available as a signal from the neck proprioceptors *x*_pro_, resulting in an indirect representation of the HIS, written as HISi; an indirect representation of the BIS, BISi, can be constructed similarly.

The latter indirect paths are

(5) $$\begin{array}{l} \text{BISi}=x_{com}-x_{pro} \end{array}$$

with mean and variance

(6) $$\begin{array}{l} \mu_{\text{BISi}}=\mu_{com}-\mu_{pro} \end{array}$$

(7) $$\begin{array}{l} \sigma_{\text{BISi}}^{2}=\sigma_{com}^{2}+\sigma_{pro}^{2}\;, \end{array}$$

and

(8) $$\begin{array}{l} \text{HISi}=x_{som}+x_{pro} \end{array}$$

with mean and variance

(9) $$\begin{array}{l} \mu_{\text{HISi}}=\mu_{som}+\mu_{pro} \end{array}$$

(10) $$\begin{array}{l} \sigma_{\text{HISi}}^{2}=\sigma_{som}^{2}+\sigma_{pro}^{2}. \end{array}$$

An estimate of the HIS, written as $\hat{\text{HIS}}$, can be constructed as a convex combination of the direct and indirect representations. Given that all variables are normal distributed, the $\hat{\text{HIS}}$ is again also normal distributed, with mean and variance

(11) $$\begin{array}{l} \mu_{\hat{\text{HIS}}}=\omega_{\text{HISd}}\mu_{\text{HISd}}+\left(1-\omega_{\text{HISd}}\right)\mu_{\text{HISi}} \end{array}$$

(12) $$\begin{array}{l} \sigma_{\hat{\text{HIS}}}^{2}=\omega_{\text{HISd}}^{2}\sigma_{\text{HISd}}^{2}+{\left(1-\omega_{\text{HISd}}\right)}^{2}\sigma_{\text{HISi}}^{2}. \end{array}$$

In these equations, ω_HISd_ is the weight for the direct representation of the HIS; the weight for the indirect representation of the HIS ω_HISi_ is equal to (1 − ω_HISd_) such that the weights sum to one. An estimate of the BIS, $\hat{\text{BIS}}$, can be constructed similarly.

Responses *r* on the RFT task are assumed to reflect the $\hat{\text{HIS}}$ after changing the sign. The mean of these responses is therefore $-1\times \mu_{\hat{\text{HIS}}}$ and the standard deviation is the same as that of $\hat{\text{HIS}}$. In the SPV task, participants choose a platform tilt stimulus that together with the AR and neck tilt stimuli ultimately results in a percept of an upright body. The mean of the response distribution is found by setting the equations for the $\hat{\text{BIS}}=0$ and solving for *x*_*som*_. This yields the following expression for the mean:

(13) $$\begin{array}{l} \mu_{\text{SPV}}=\left(\frac{\omega_{\text{BISd}}-1}{\omega_{\text{BISd}}}\right)\mu_{\text{BISi}}. \end{array}$$

Note that when ω_BISd_ = 1, the mean is 0, corresponding to the gravitational vertical. The standard deviation is equal to that of the somatosensory system σ_*som*_.

In the model proposed by Clemens et al. (2011), the weights attributed to direct and indirect representations are defined by the variances of the direct and indirect paths, reflecting the theory that perception is consistent with the mechanisms of Bayesian inference, or more simply MLE (Ernst and Banks, 2002; Hillis et al., 2002; Ernst and Bülthoff, 2004). To evaluate the tenability of the model, we compare how well experimental data can be explained by three versions: “fixed,” where ω_HISd_ and ω_BISd_ have fixed values of 1—this reflects the hypothesis that representations of the HIS and BIS depend on direct paths only, and allows us to test whether there is evidence for interactions between direct and indirect paths at all; “free,” where ω_HISd_ and ω_BISd_ are free parameters, constrained to the range (0, 1), which reflects the hypothesis that there are indeed interactions between direct and indirect representations, but their weightings are not proportional to signal variances and therefore not consistent with MLE; and finally “MLE,” where ω_HISd_ and ω_BISd_ are calculated on the basis of the variances of the sensory signals, as predicted by MLE:

(14) $$\begin{array}{l} \omega_{\text{HISd}}=\frac{\sigma_{\text{HISi}}^{2}}{\sigma_{\text{HISd}}^{2}+\sigma_{\text{HISi}}^{2}} \end{array}$$

(15) $$\begin{array}{l} \omega_{\text{BISd}}=\frac{\sigma_{\text{BISi}}^{2}}{\sigma_{\text{BISd}}^{2}+\sigma_{\text{BISi}}^{2}}. \end{array}$$

### Data Analysis

Prior to the actual data analyses, training trials (72, combined over participants), trials where participants reported to have made a mistake (3; e.g., “pressed button too soon”), and trials where there was an issue with COP data quality (10) were discarded. Data removed based on these criteria totaled 4.2% (85 of 1,983 trials). A mean correction was applied by subtracting the mean value observed in the head upright conditions of the RFT and SPV tasks from all responses; in both the head upright and head tilted conditions within each participant. This procedure corrected for systematic biases (offsets) due to for instance posture or tilt of the head-mounted display relative to the head, and ensured that data were symmetric around 0° in head upright conditions. Not correcting for these sources of error would inflate estimated variances (Alberts et al., 2016).

To assess the effects of experimental manipulations without imposing the structure of the verticality perception model, we used LME models. These are regression models with platform, neck, and AR tilt as categorical predictors, at the group (fixed effects) and individual level (random effects). These models do not impose any constraints on the shape of the relation between the independent and dependent variables other than that the score in each condition has a certain mean and variance, and also account for individual variability in the value of the coefficients. In other words, these models allow us to evaluate whether there are differences between conditions, much in the same way as the familiar *t*-test or one-way ANOVA would. For the responses *r* on the RFT and SPV tasks, the model took the following form (in Wilkinson notation; Wilkinson and Rogers, 1973):

(16) $$\begin{array}{l} r\sim 1+\varphi_{\text{P}}+\varphi_{\text{N}}+\varphi_{\text{AR}}+\left(1+\varphi_{\text{P}}+\varphi_{\text{N}}+\varphi_{\text{AR}}|\text{id}\right). \end{array}$$

In this equation, 1 represents an intercept, and id participant id. The variables outside the brackets represent the fixed effects; variables inside the brackets represent the random effects (i.e., variability added to the fixed effects at the individual level). Since this part of the analysis is aimed at assessing general effects, we will only consider the fixed effects.

The LME models provide descriptions of the data, but do not explain how perception of verticality is achieved by the brain. For this purpose, we consider the verticality perception model (Figure 3). The perception model specifies how tilt stimuli result in different internal representations of verticality. This model is much simpler than the LME models in the sense that it has only five parameters (φ_*N*_, σ_*som*_, σ_*pro*_, σ_*com*_0__, *K*_*com*_), but it is also less flexible in explaining the data than the LME models (in general) due to the imposed relations between variables. If we find that the perception model nevertheless explains the data well, this can be interpreted as evidence that the model captures the nature of verticality perception. As detailed in the Verticality Perception Model section, we fitted three versions of this model: one version where indirect effects were effectively excluded by fixing their weights to zero; one version where the weights attributed to the indirect effects were free parameters; and one version where the weights were set according to predictions by a MLE framework. The model was fitted to the joint RFT and SPV responses for each participant individually.

We evaluated which model provides the best account of the data by comparing the Bayesian Information Criterion (BIC) fit index for each model. The BIC is a score that expresses relative model quality, and can be used for model selection. It is based on the model likelihood and includes a penalty for the number of parameters in the model (Schwarz et al., 1978). The model with the lowest BIC score is considered the best in an absolute sense. Differences in model BIC scores (ΔBIC) between 0 and 2; 2 and 6; 6 and 10 are considered negligible, positive, and strong evidence, respectively, and ΔBIC > 10 are considered decisive evidence (Kass and Raftery, 1995).

## Results

### Analysis of Perception Data

**Figure 4:**
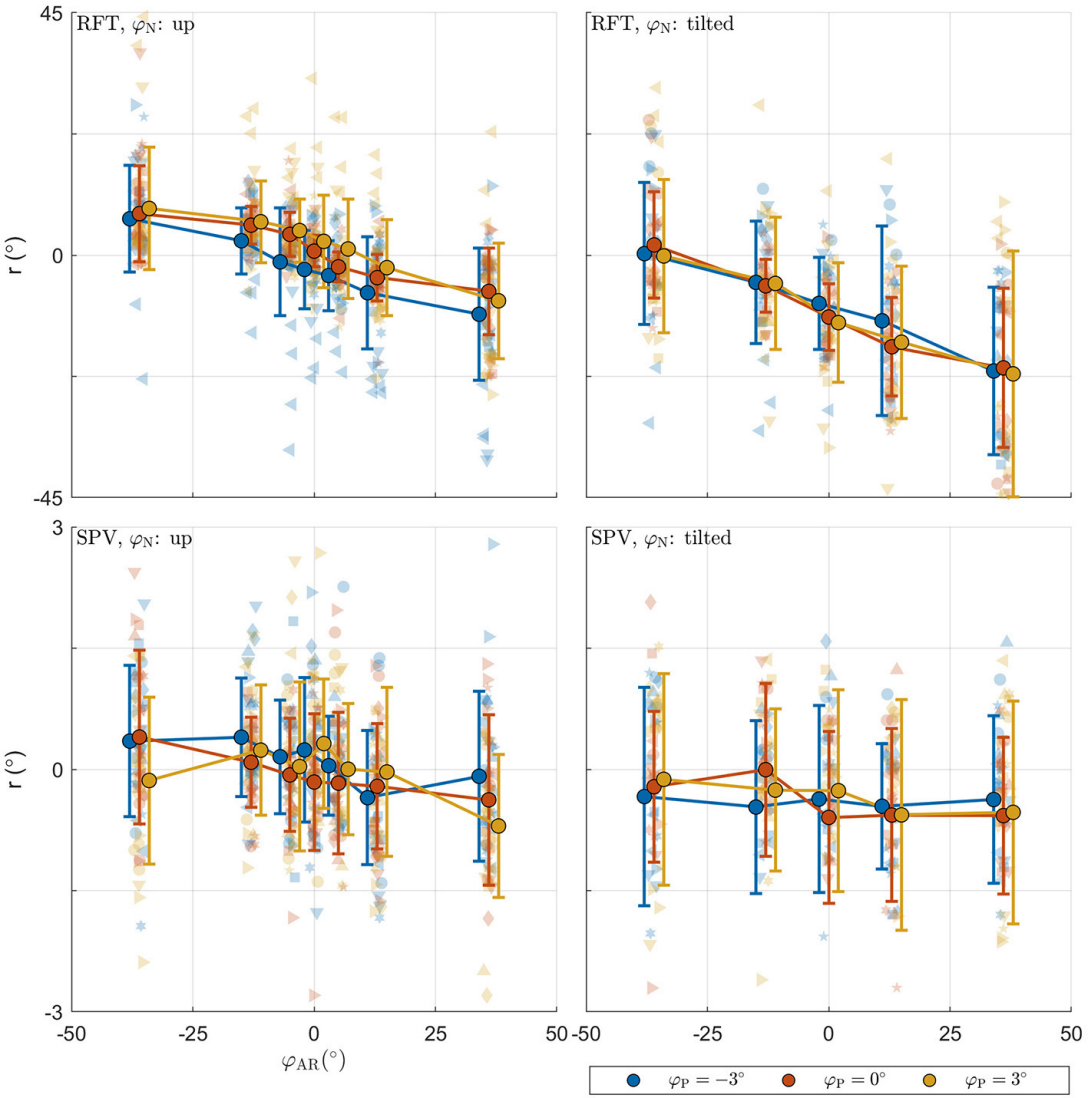
Raw perception data collected in the experiment. For each panel, the row specifies the task (SPV, RFT); the column specifies neck tilt (head upright, head tilted). The x-axis specifies the AR system tilt angle, the y-axis the response. Data colored in blue, orange, and yellow represent observations for platform tilts of −3, 0, 3°, respectively. Individual responses *r* are represented by semi-transparent points. In the RFT task, *r* is the recorded rod setting; in the SPV task, *r* is the recorded platform tilt setting. Each participant has a different marker shape. The opaque dots, connected by lines, represent the average for each condition. Errorbars represent ±1 standard deviation.

The raw data collected in the experimental tasks is shown in Figure 4. For each panel, the row specifies the task (RFT, SPV) and the column specifies the neck tilt (head upright, head tilted). Patterns in the data appear consistent with expectations. The panels with the data from the RFT task reveal a negative relation between response bias and AR system tilt, with distinct additional offsets when the head or platform are tilted. For the extreme values of φ_AR_, biases of approximately ±10° are observed. The panels with the SPV data also suggest a negative trend, albeit much smaller, and without offsets for head or platform tilt. In this task, biases of approximately ±0.25° are observed for the extreme values of φ_AR_.

The coefficients obtained in fitting the LME model are presented in Table 1.

**Table 1:** Estimated coefficients (°) for the parameters in the LME model of perception data.

	**RFT** **task**			**SPV** **task**		
**Variable**	**Coefficient**	***t*_**(966)**_**	**p**	**Coefficient**	***t*_**(912)**_**	**p**
Intercept	8.357	4.406	0.000	0.253	3.048	0.002
φ_N_ = tilted	−10.303	−6.716	0.000	−0.380	−1.613	0.107
φ_AR_ = −13	−4.233	−5.046	0.000	0.013	0.132	0.895
φ_AR_ = −5	−6.737	−6.705	0.000	−0.147	−1.278	0.202
φ_AR_ = 0	−9.266	−11.045	0.000	−0.117	−1.204	0.229
φ_AR_ = 5	−10.689	−10.643	0.000	−0.229	−1.778	0.076
φ_AR_ = 13	−13.699	−8.618	0.000	−0.364	−2.769	0.006
φ_AR_ = 36	−18.719	−7.480	0.000	−0.464	−3.240	0.001
φ_P_ = 0	1.123	0.627	0.531	−0.116	−1.724	0.085
φ_P_ = 3	1.078	0.299	0.765	−0.081	-0.628	0.530

*The reference condition is φ_N_ = up, φ_AR_ = −36, φ_P_ = −3, with an average score equal to the intercept. Averages for other conditions are given by the sum of the intercept and coefficients that specify that condition. For example, the average value in the RFT task condition where {φ_N_ = tilted, φ_AR_ = 36, φ_P_ = 0} = 8.357 + (−10.303) + (−18.719) + 1.123 = −19.542, which can be verified in the top right panel of Figure 4*.

For the RFT task, an ANOVA showed that effects of neck and AR system tilt were significant [φ_N_: *F*_(1,966)_ = 45.106, *p* < 0.001; φ_AR_: *F*_(6,966)_ = 38.202, *p* < 0.001]. The coefficient for φ_N_ was −10.303. This indicates that in the conditions with the head tilted clockwise, rod settings were tilted counterclockwise. The value of approximately −10° is plausible as average head tilt. The coefficients for the levels of φ_AR_ describe a negative slope. This indicates that rod settings were biased in the direction of the vertical expressed in the visual scene. The ANOVA was not significant for the effect of platform tilt φ_P_ [*F*_(2,966)_ = 0.236, *p* = 0.789].

For the SPV task, only the effect of AR tilt was significant [φ_AR_: *F*_(6,912)_ = 3.353, *p* = 0.003]. The coefficients for the levels of φ_AR_ again indicated a negative trend. This is consistent with expectations: if the $\hat{\text{BIS}}$ is constructed as a convex combination with positive weights, a response *r* = 0° can only be achieved by combining a positive with a negative value.

The model BIC scores were 6999.169 for the RFT task, and 2500.317 for the SPV task. The combined BIC score, for comparison with the perception-model analysis, was 9528.615.

### Analysis of Postural Data

**Figure 5:**
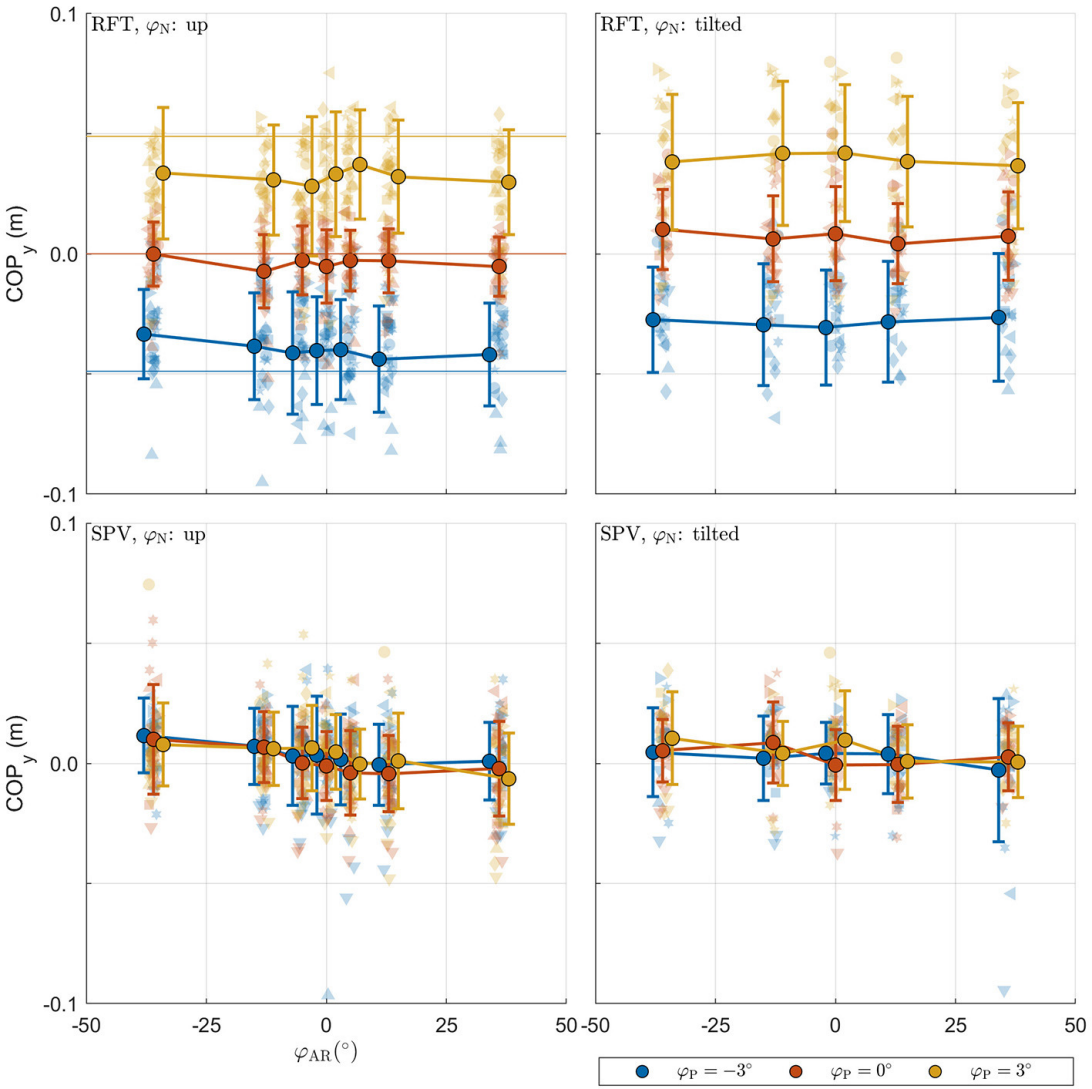
Raw postural data collected in the experiment. For each panel, the row specifies the task (SPV, RFT); the column specifies neck tilt (head up, head tilted). The x-axis specifies the AR system tilt angle, the y-axis the response. Data colored in blue, orange, and yellow represent observations for platform tilts of −3°, 0°, 3°, respectively. Individual observations are represented by semi-transparent points. Each participant has a different marker shape. The opaque dots, connected by lines, represent the average for each condition. Errorbars represent ±1 standard deviation.

The raw postural data collected in the experiment is shown in Figure 5. The panels with the data from the RFT task reveal that the COP shifts in the expected direction with platform tilt, with a slight additional offset for neck tilt φ_N_. These effects appear to be absent in the panels for SPV data. This is expected as platform tilts here serve only as initial positions. SPV data do however suggest a negative trend for camera tilt.

An assumption made in the modeling is that participants' bodies are rigid relative to the platform, and therefore that the platform tilt stimulus can be used as the mean (i.e., it is unbiased) of the somatosensory signal. Because participants stood freely on the platform, it is however possible that they counteracted this tilt. The validity of the assumption was therefore tested using COP measurements from the RFT task, head upright condition. We approximated the height of each individual's center of mass as half their length (Loomis, 2011), and determined how much the COP of a rigid body with the center of mass at this height would shift for each platform tilt. These expected values were compared to measured values using a linear mixed effects model of the form: COP_y,measured_ ~ COP_y,expected_+(1+COP_y,expected_|id) This model yielded a slope of 0.731 for the fixed effect of COP_y,expected_. This value is below 1 [*F*_(1,642)_ = 4.858, *p* = 0.028], and indicates that participants counteracted platform tilts by about 27%. The assumption was thus violated. To account for this, we also fitted the perception models using individually corrected values for tilt stimuli. Although this procedure affected the estimated coefficients, it did not lead to any different conclusions in model comparisons. For simplicity, we therefore ultimately chose to use the platform tilt values as stimuli in the analysis presented below.

Analogous to the analysis of perception data, we evaluated whether the experimental manipulations resulted in systematic changes in COP_y_ for both tasks, using an LME model of the same form. The estimated coefficients are given in Table 2.

**Table 2:** Estimated coefficients (cm) for the parameters in the LME model of postural data (COP_y_).

	**RFT**			**SPV**		
**Variable**	**Coefficient**	***t*_**(964)**_**	**p**	**Coefficient**	**t_**(911)**_**	**p**
Intercept	−3.726	−7.978	0.000	0.905	2.210	0.027
φ_N_ = tilted	1.047	2.366	0.018	0.056	0.150	0.881
φ_AR_ = −13	−0.250	−1.665	0.096	−0.268	−1.522	0.128
φ_AR_ = −5	−0.352	−1.592	0.112	−0.507	−2.227	0.026
φ_AR_ = 0	−0.221	−0.955	0.340	−0.560	−3.060	0.002
φ_AR_ = 5	−0.050	−0.253	0.800	−0.932	−3.388	0.001
φ_AR_ = 13	−0.338	−1.263	0.207	−0.887	−4.871	0.000
φ_AR_ = 36	−0.342	−1.419	0.156	−1.014	−4.473	0.000
φ_P_ = 0	3.588	6.318	0.000	−0.196	−1.027	0.305
φ_P_ = 3	6.987	6.125	0.000	0.012	0.067	0.947

*The reference condition is φ_N_ = up, φ_AR_ = −36, φ_P_ = −3, with an average score equal to the intercept. Averages for other conditions are given by the sum of the intercept and coefficients that specify that condition*.

For the RFT task, the effects for φ_P_ and φ_N_ were significant [*F*_(2,964)_ = 20.286, *p* < 0.001 and *F*_(1,964)_ = 5.597, *p* = 0.018, respectively]. The coefficients for the different levels were positive and increased in magnitude, indicating that the COP shifted in the expected direction. There was no effect of φ_AR_[*F*_(6,964)_ = 0.982, *p* = 0.436]. This was unexpected and contrasts with the findings for the perception data.

For the SPV task, only the effect of φ_AR_ tilt was significant [*F*_(6,911)_ = 5.516, *p* < 0.001]. The coefficients for the levels of φ_AR_ indicated a negative trend. Positive values of AR tilt result in a visual stimulus that suggests clockwise head tilt. A negative trend thus indicates that participants shifted their weight to counteract the visually perceived tilt. Between the extremes of $\varphi_{\text{AR}}=\pm 36^{{}^{\circ}}$, the COP_y_ shifts 1.014 cm (i.e., the regression coefficient for $\varphi_{\text{AR}}=36^{{}^{\circ}}$). This means that 36° of visual tilt causes the COP to shift by approximately 0.5 cm, which translates to 0.3° body tilt for a center of mass at 0.93 m. The absence of an effect for φ_P_ is explained by the fact that platform tilt only served as a starting point.

### Verticality Perception Model Fits

To explain observations of different sensor weightings between studies using different experimental tasks to probe verticality perception, we evaluate a model where percepts are constructed from direct estimates of the HIS and BIS based on signals from head and body sensors, and indirect estimates based on signals from body and head sensors corrected for neck tilt (Figure 3).

We fitted three versions of this model: one version where indirect effects were effectively excluded by fixing their weights to zero (“fixed”); one version where the weights attributed to the indirect effects were free parameters (“free”); and one version where the weights were set according to predictions by a MLE framework (“MLE”). We evaluated which version provides the best account of the data by comparing fit indices. To determine whether the models provide a good account of the data in general, we also compared their fit to that of the LME models.

The BIC scores for the “fixed,” “free,” and “MLE” versions of the model were 10532.188 (median *R*^2^ = 0.413), 9436.852 (median *R*^2^ = 0.538), and 9325.481 (median *R*^2^ = 0.540), respectively. This means that the MLE model provided the best fit. Compared to the next-best model (“free”), the evidence in favor of the MLE model was ΔBIC = 111.371. ΔBIC > 10 may be considered decisive evidence (Kass and Raftery, 1995). Note that this is not a reflection of the small increase in *R*^2^ *per se*, but more so that the model achieves this fit despite the omission of the free weighting parameters. On an individual level, the MLE model provided the best fit for 8/10 participants, with an average ΔBIC = 10.577 (range: 3.471 − 26.697). For the remaining two participants, the data were best described by the model where the weights for the direct and indirect representations were free to vary (ΔBIC = 8.840 and 10.491).

**Figure 6:**
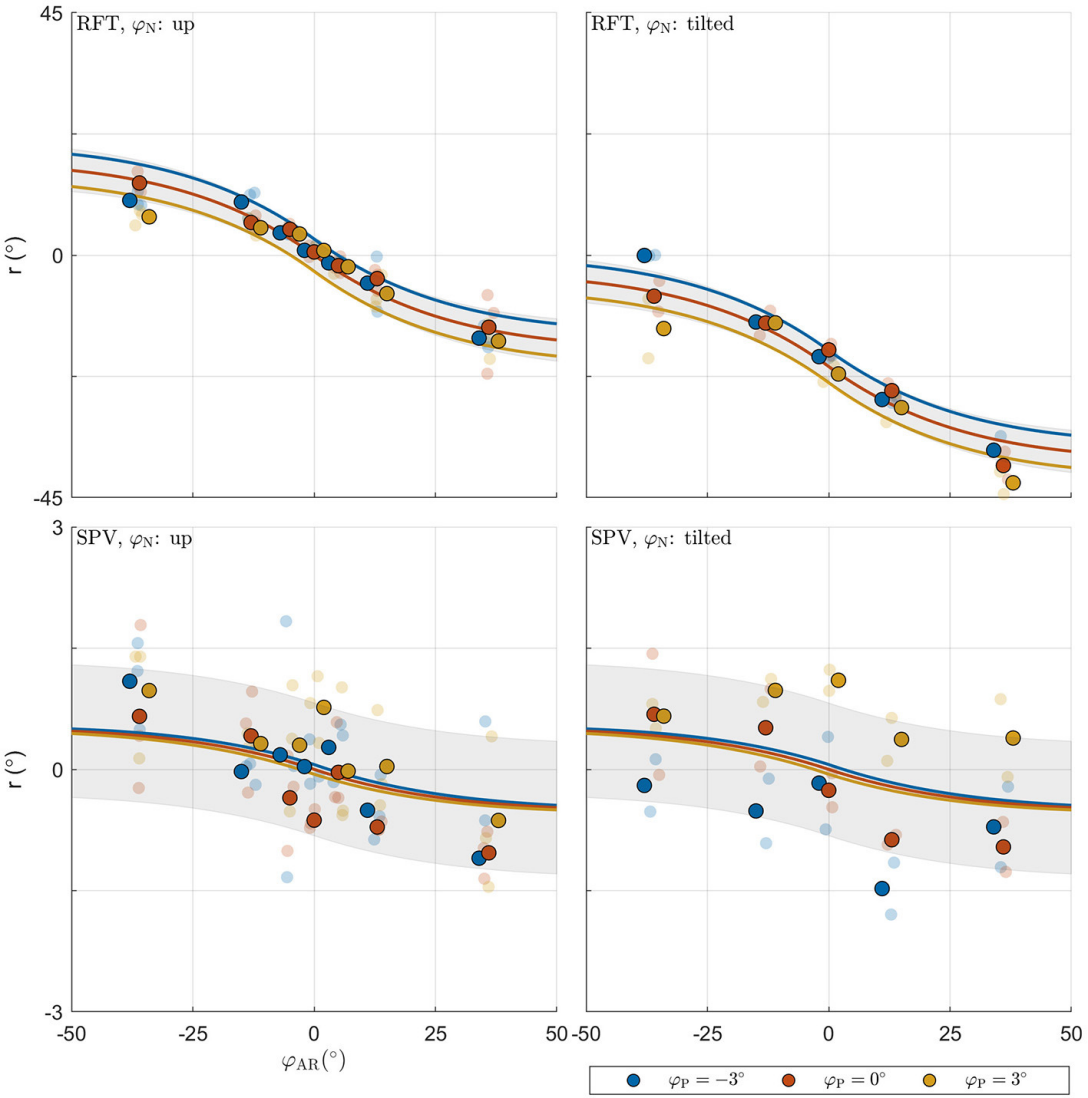
Illustration of model fit for an example participant. For each panel, the row specifies the task (RFT, SPV); the column specifies neck tilt (head up, head tilted). Data colored in blue, orange, and yellow represent observations for platform tilts φ_P_ of −3, 0, 3°, respectively. Individual observations are represented by semi-transparent points. The x-axis specifies the AR system tilt angle, the y-axis the response angle. The lines represent the predicted mean response, with colors matching the φ_P_ condition. The shaded areas represent ±1 standard deviation predicted for $\varphi_{\text{P}}=0^{{}^{\circ}}$ conditions.

The combined BIC score of the LME models was 9528.615. Comparison of this value with the score for the MLE verticality perception model (9325.481) also favors the latter, and indicates that this model provides a more parsimonious account of the data. Figure 6 shows the fit of the model for an example participant.

The estimated coefficients and model *R*^2^ goodness-of-fit metrics for each participant are given in Table 3. The median value for head tilt φ_N_ was 9.416. This value differed between individuals because head tilt was self-imposed by participants. Differences arose due to the placement of the support on their shoulders and the length of the neck. The median standard deviations σ_*som*_, σ_*pro*_, σ_*com*_0__ were 0.811, 9.276, and 8.572. The standard deviation of the signals from the combined head sensors furthermore increased by 0.249° per degree AR tilt.

**Table 3:** Verticality perception model coefficients and *R*^2^ fit indices.

**Participant id**	**φ_N_**	**σ_*som*_**	**σ_*pro*_**	**σ_*com*_0__**	***K*_*com*_**	***R*^2^**
1	4.713	0.880	11.203	5.882	0.356	0.389
2	9.598	0.745	3.735	3.500	0.237	0.737
3	14.990	0.993	24.056	11.703	0.330	0.500
4	8.592	0.611	22.471	26.488	0.376	0.018
5	3.684	1.154	9.784	10.296	0.392	0.259
6	20.677	0.821	4.690	3.433	0.072	0.939
7	12.673	0.801	8.769	3.958	0.219	0.792
8	12.598	0.762	11.206	6.848	0.262	0.649
9	9.233	0.786	6.403	10.483	0.094	0.580
10	6.578	1.840	4.594	21.113	0.000	0.180
Median	9.416	0.811	9.276	8.572	0.249	0.540
SD	5.099	0.349	7.168	7.789	0.138	0.277

In the MLE model, the standard deviations determine the weight attributed to the internal representations in the formation of the estimates $\hat{\text{HIS}}$ and $\hat{\text{BIS}}$. The median weight for the direct representation of the BIS ω_BISd_ was 0.997, meaning that the indirect representation BISi has only a very small effect on the perception of the BIS (ω_BISi_ = 1−ω_BISd_). Variation of this weight, due to variation of the visual noise with AR tilt, was also small, ranging between ω_BISd_ = 0.996 for $\varphi_{\text{AR}}=0^{{}^{\circ}}$ and ω_BISd_ = 0.998 for $\varphi_{\text{AR}}=\pm 36^{{}^{\circ}}$. In a practical sense, this implies that the body-centered percept of verticality is almost entirely determined by somatosensory information. Conversely, the median weight ω_HISd_ for the direct representation of the HIS was 0.383, meaning that the indirect representation HISi surprisingly has a stronger effect on the perception of the HIS than the direct representation HISd. This weight also varied considerably depending on AR tilt, ranging between 0.541 for $\varphi_{\text{AR}}=0^{{}^{\circ}}$ and 0.220 for $\varphi_{\text{AR}}=\pm 36^{{}^{\circ}}$. The latter findings imply that the weight attributed to somatosensory information increases when visual information deviates from upright (see also section 4). Overall, the weights show that the head centered representation of verticality is determined about equally by somatosensory information on the one hand, and combined information from the visual and vestibular systems on the other. Note how the sigmoidal effect in the responses on the experimental tasks is explained through the dependency of σ_*com*_ on φ_AR_. Since σ_*com*_ is assumed to increase linearly with φ_AR_, the weighting factors ω_HISd_ and ω_BISi_ decrease for larger values of φ_AR_. This creates a nonlinear effect, even though the verticality perception model is basically linear (Figure 3).

Two participants showed particular idiosyncrasies that could be addressed by including additional effects. For participant with id 4, a much improved fit was obtained by allowing a negative gain (*K*_*som*_) on the platform tilt (*R*^2^ = 0.799 vs. *R*^2^ = 0.018). The postural data did not differ notably from other participants, suggesting that this person misperceived platform tilt. Including this gain yielded the following coefficients: φ_N_ = 8.657, σ_*som*_ = 0.610, σ_*pro*_ = 9.560, σ_*com*_0__ = 10.292, *K*_*com*_ = 0.187, *K*_*som*_ = −5.789. Notable changes are coefficients σ_*pro*_, σ_*com*_0__, *K*_*com*_, which are much closer to the sample median when this gain is included. For participant id 10, the fit was improved by including an additional offset for neck tilt in the SPV task (φ_N-SPV_; *R*^2^ = 0.210 vs. *R*^2^ = 0.180). Including this gain yielded the following coefficients: φ_N_ = 6.578, σ_*som*_ = 1.242, σ_*pro*_ = 4.779, σ_*com*_0__ = 20.376, *K*_*com*_ = 0.000, φ_N-SPV_ = −2.402. This resulted in a considerably lower σ_*som*_ coefficient. Figures showing the data and obtained fits for all individual participants are available in the Supplementary Material.

## Discussion

The study presented here was designed to determine how visual information is combined with somatosensory and proprioceptive information to produce percepts of the HIS and BIS. We presented participants with various (incongruent) visual-inertial tilt stimuli, using a motion base and an AR setup that allowed independent manipulation of the visually perceived roll tilt angle of their actual surroundings. We probed perception of the BIS using an SPV task, and perception of the HIS using an RFT task. The perception data shows clear effects of body tilt, head tilt, and visual tilt in both experimental tasks. For the RFT task, it was found that visual tilt biased responses in the direction of the vertical expressed in the visual scene; for the SPV task, it was observed that participants adjusted platform tilt to correct for illusory body tilt induced by the visual stimuli. Effects were much larger in the RFT task than in the SPV task. We find that a verticality perception model based on principles of statistical optimality can provide a parsimonious account of the findings including non-linear effects. In the following, we discuss our findings in relation to the literature.

### Rod and Frame Test

The RFT was first developed to study the determinants of orientation perception (Witkin and Asch, 1948). Since its introduction, many studies have demonstrated that there is a periodical relation between visual frame tilt and biases in the alignment of a visual rod with the subjective vertical, with a periodicity of about 45° and peak biases in the order of 1−10° (Witkin and Asch, 1948; Wenderoth, 1974; Cian et al., 1995; Alberts et al., 2016). This bias is known as the rod-and-frame effect, and has been attributed to interactions between vision and other senses in the construction of a percept of the HIS (Mittelstaedt, 1995).

Vingerhoets et al. (2009) investigated these interactions using a variant of the RFT task where participants adjusted the orientation of a luminous line to indicate verticality, against the background of a single other, tilted, luminous line. Participants were seated in a tilting chair that was used to manipulate body tilt to {0, 60, 120}°, and were shown the background line at tilt angles between −90:90°, in 10° steps. They found that for small tilts (i.e., up to 45°), responses were biased in the direction of the line. Although exact values were not reported, the difference between peak responses for clockwise and counterclockwise frame tilts suggested a maximum bias of about 2°, at line tilts between 15−20° for upright participants. This value increased to about 15° for physically tilted participants. Alberts et al. (2016) elaborated on the work of Vingerhoets et al. (2009), using a forced-choice version of the RFT task. On a large series of experimental trials, participants were presented with square visual frames tilted between −45:40°, in 5° steps; either with the head upright or with the head tilted 30° to the right. For each trial, participants judged whether a visual rod presented at a range of angles was tilted clockwise or counter clockwise. The authors fitted cumulative Gaussian functions to the data, and interpreted the mean and standard deviation as measures of perceptual bias and precision. The results were consistent with those of Vingerhoets et al. (2009), showing a maximum bias of approximately 2° with the head upright, and a slight increase of the bias with the head tilted.

In comparison, the present data show a maximum bias of ±9.360° at $\varphi_{\text{AR}}=\pm 36^{{}^{\circ}}$. This is considerably larger than in the above studies^1^. The differences in magnitude of the peak bias and the associated visual tilt angle between the present and earlier studies (Vingerhoets et al., 2009; Alberts et al., 2016) could be related to the use of the AR system. It has been shown that a reduction of the size of a visual frame reduced its biasing effects in the RFT task (Cian et al., 1995; Alberts et al., 2016). Compared to a luminous line or frame, the present visual stimulus is considerably “richer”: it contains polarity information, support relationships between objects, and shows motion of the experimenter (e.g., Howard, 1982; Howard et al., 1990; Oman, 2003; Harris et al., 2011). The stimulus can thus be considered to provide a frame that spans the entire field of view, which may increase the weight attributed to the visual signal. The difference in the visual tilt angle where the peak bias is observed may be related to differences in the stimulus' periodicity. For rectangular frames and single lines (which can be interpreted as horizontal or vertical), the periodicity is 90°, which is a reduction of a factor four compared to the present stimulus (360°). This could explain why the peak biasing effect for a frame occurs at a smaller visual tilt angle than for the present stimulus.

### Subjective Postural Vertical

Tasks that measure the SPV have been developed to characterize perception of the BIS. SPV tasks are used in clinical settings to determine the extent of damage to the vestibular end organs or of more central vestibular lesions (Clark and Graybiel, 1963; Bisdorff et al., 1996; Perennou et al., 1998). As such, these tasks are usually performed either under conditions of complete darkness or with eyes open, but without manipulation of visual tilt.

In static implementations of the task, participants are seated in a tilting chair and are asked to adjust its orientation to match certain subjective reference tilts (e.g., Earth-vertical), or they are passively tilted to certain target angles and provide forced-choice judgments on whether their position is clockwise or counter clockwise relative to a subjective reference. In dynamic implementations, participants are oscillated around the vertical and asked to indicate the moment when they perceive themselves to be upright (Mann et al., 1949; Mittelstaedt, 1995; Clemens et al., 2011). For normal control subjects, the static SPV is unbiased and has a variability of 2 − 3.5° for near-upright positions (e.g., Mann et al., 1949: 1.9°; Clemens et al., 2011: 3.3°). For dynamic tasks, biases have reported in the direction of motion, suggesting perception delays, and the variability increases to 3 − 6° (e.g., Mann et al., 1949: 3.2°; Bisdorff et al., 1996: 5.9°).

In a recent study by our group (Nestmann et al., 2020), we explicitly evaluated effects of vision on the SPV. In this previous study, the same general apparatus and experimental paradigm were used as in the present study, with the exception that participants were seated in a bucket seat that was mounted to the platform, instead of standing freely. There, we found biases between 1 − 3° for visual tilt angles of ±36°, with the larger biases observed for older participants. The present findings match those of the previous study in terms of direction, but here the effect of visual stimuli was smaller, with a difference between the peak biases at $\varphi_{\text{AR}}=\pm 36^{{}^{\circ}}$ of 0.464°. The residual standard deviation of the LME model was 0.835°, which is in line with previous observations of variability in responses. One possible explanation for the reduced effect size may be that participants were standing and partially counteracted platform tilts, as was found in the analysis of the postural data. It is also possible that the noise in the somatosensory signal is smaller during standing than while seated because posture maintenance provides continuous feedback including forces sensed in the legs. This would result in a larger weight of the direct estimate of the BIS in the present study.

### Comparison of Perceptual and Postural Effects

Past research has consistently shown that vision reduces postural sway in dynamic settings (Lee and Aronson, 1974; Lee and Lishman, 1975; Lestienne et al., 1977; Bronstein, 1986; Van Asten et al., 1988; Dijkstra et al., 1994; Golomer et al., 1999), and also reduces variance of the COP in static conditions (Isableu et al., 1997). Based on such observations, we expected that perceptual biases in the RFT and SPV tasks would be associated with shifts of the COP.

We were therefore surprised to find that visual tilt stimuli did not appear to affect posture in the RFT task whereas sizable perceptual biases were found. However, the lack of similarity between perceptual biases and the COP could be a reflection of the dissociation between the HIS and BIS: the RFT task reflects perception of the HIS, whereas postural control is arguably more closely related to perception of the BIS. Indeed, the data obtained in the SPV task do show a negative bias that is accompanied by a similar postural effect. Moreover, in the RFT task, platform tilt angles were maintained for the duration of a trial. For ±3° platform tilts, this means that participants had to exert more effort to maintain their posture during a trial than in the SPV task, where participants adjusted the platform to an upright position. As a consequence, participants may have been more unstable in the RFT task. This increases variability in the data, and may have occluded effects of the visual stimulus. Indeed, the coefficients for the effects of platform and head tilt on the COP_y_ in the RFT task are about an order of magnitude larger than the effects of visual tilt, which is not the case in the SPV task, where the visual effects are the most pronounced. This may mean that the relatively large effects of platform and head tilt on posture in the RFT task may have occluded the much smaller effects of vision, which were subsequently lost in noise.

The effect of visual tilt on posture in the SPV task can be derived from the difference between the COP_y_ positions at the extremes of $\varphi_{\text{AR}}=\pm 36^{{}^{\circ}}$. Doing so shows that 36° of visual tilt shifts the COP by about 0.5 cm, which corresponds to 0.3° body tilt. This value is very similar to findings by Isableu et al. (1997), who found that participants who were selected for a high sensitivity to rod-and-frame effects tilted their bodies on average 0.4° in response to 18° tilts of a simple visual frame.

### Evaluation of the Verticality Perception Model

The primary goal of this study was to determine whether differences in the apparent weight attributed to visual stimuli between tasks that probe verticality perception in different ways can be explained by different tasks relying either on percepts of the HIS or BIS. To assess how the responses depend on the experimental manipulations without imposing the structure of the verticality perception model, we first evaluate the results of the LME model. These models can be likened to the familiar ANOVA in that they allow us to evaluate whether differences exist between conditions. Based on these models, we conclude that (1) visual information affects responses on both tasks, and that (2) the effect of vision is much larger for the RFT task. A comparison between fit indices favored the MLE verticality perception model over the LME models. This provides evidence that (3) it is plausible that responses for both tasks derive from different verticality percepts. Moreover, comparisons between different versions of the perception model indicate that (4) verticality percepts are constructed from both direct and indirect internal representations of the HIS or BIS, and that (5) the weights attributed to the internal representations are consistent with predictions of statistically optimal perception models (Ernst and Banks, 2002; Hillis et al., 2002; Ernst and Bülthoff, 2004).

The MLE verticality perception model is an adaptation of the model by Clemens et al. (2011). The model accounts for differences in the strength of direct and indirect effects in the construction of different percepts through the relative size of signal variances (noises) and the directionality of the effects: the weights are inversely proportional to the variances of the signals, such that less noisy signals receive the most weight. As a specific example, when all sensors signal being upright (0°), the somatosensory signal has a small standard deviation (median: σ_*som*_ = 0.811) and the neck and head tilt signals have a large standard deviations (medians: σ_*pro*_ = 9.276 and σ_*com*_0__ = 8.572). In the construction of a percept of the BIS, the somatosensory signal receives a large weight because its variance is small (ω_BISd_ = 0.997); the indirect pathway has a variance equal to the sum of the head and neck tilt variances, which is much larger and therefore has very little effect (ω_BISi_ = 0.003). Conversely, in the construction of a percept of the HIS, the signal variances are the same, but result in different weights because the signals combine differently: in upright conditions, the direct head tilt signal is weighted slightly more (ω_HISd_ = 0.541) than the indirect somatosensory signal (ω_HISd_ = 0.459) because the correction for neck tilt results in a larger variance.

Our model differs from the Clemens et al. (2011) model in two ways. The primary difference is that our model does not include priors. In proper Bayesian models, priors account for effects of internalized knowledge on perception. Here, the priors can be included in the model as additional inputs to the weighted sums that result in the HIS and BIS. Although we do not object to the notion that internalized knowledge can affect perception, we chose not to include priors here: first, studies have found no evidence that the BIS is affected by prior knowledge (Mittelstaedt, 1995; Bortolami et al., 2006; Clemens et al., 2011; Medendorp et al., 2018); second, whereas there is evidence that the visual vertical is affected by an “idiotropic vector” (Mittelstaedt, 1983), which represents internalized knowledge that the vertical is usually aligned with the long body axis, it is not possible to actually estimate its effect in the present paradigm. The reason for this is that this prior and the somatosensory signal both approximately align with the long body axis, meaning that it is not possible to uniquely estimate their relative contributions. In an attempt to facilitate comparison between the present model and the Clemens et al. (2011) model, we tried to fit a version of the model that did include this prior, but with a fixed standard deviation [values of 6.5° (Alberts et al., 2016) and 12.5° (Clemens et al., 2011)]. However, the optimizations would not converge properly for this variation of the model, as in this case the standard deviation of the proprioceptive signal on neck tilt tended to approximate the bounds. When the standard deviation of the prior was set as a free parameter, either the standard deviation of the neck tilt or the prior would approximate the bounds. This implies that either the prior or the indirect contribution of the somatosensory signal to the HIS was effectively removed from the equation, and reflects the fact that the prior and somatosensory signals point in nearly the same direction. A second difference is that Ocular Counter-Roll (OCR) was not accounted for. OCR is roll rotation of the eyes in the direction opposite to the inducing stimulus, which can be roll tilt stimulation of the otoliths (Miller, 1962; Cheung et al., 1992; Kingma et al., 1997) but also visual tilt, when it is known as opto-kinetic torsion (Brecher, 1934; Cheung and Howard, 1991; Farooq et al., 2004). OCR has a gain up to 10%, but this gain differs between individuals. It has been shown that the perceptual system does not correct for such torsional motion (Wade and Curthoys, 1997). Because the eyes roll in the direction opposite to the head tilt, it may be expected to cause an underestimation of the visually perceived head tilt angle. In principle, OCR can be modeled by imposing a gain on the visual tilt signal, and a fixed gain may be used when the amount of OCRis not known for an individual (Vingerhoets et al., 2009; Clemens et al., 2011). However, in the present paradigm, fixing the value of gain would be equivalent to reducing the weight of the visual signal. This means that it would not be possible to distinguish these effects. Practically, the omission of priors and OCR means that the role of vision and the amount of head tilt may be somewhat underestimated in the present verticality perception model.

A limitation of the present paradigm is that it did not allow a delineation of the interactions between visual and vestibular observations on verticality that result in the direct internal representation of the HIS, HISd. With reference to the MLE model from the literature (Ernst and Banks, 2002; Hillis et al., 2002), the HISd could itself be thought of as being constructed as a weighted sum of vestibular and visual signals with exact predictions on its mean and variance. Theoretically, it should then be possible to include these relations in the modeling, and thereby estimate the relative contributions of the visual and vestibular systems. However, we found that when doing so, the optimizations would not properly converge: either the coefficients for the variances made no sense theoretically, or the optimizations settled on the bounds set for the coefficients. We were not able to identify a single cause for these issues, but believe it to be related to interconnection of all variances through the model equations, and to additional sources of noise being present in the response data other than pure perceptual noise. Similar to including effects of the idiotropic vector or OCR, this might be dealt with by fixing the value of one of the variances, or by fixing the ratio of visual and vestibular variances consistent with observations made in other studies. However, these strategies themselves led to problems with model convergence, and extreme interpersonal differences in model coefficients. In future work, this limitation could be overcome by also collecting data in conditions where the view of the room through the head-mounted display is replaced by a black background, which would provide a baseline condition not affected by visual tilt.

Finally, it should be noted that there is an alternative explanation for the apparent sigmoidal relation between visual tilt and response bias (Figure 6). This effect is in principle well accounted for by the dependence of σ_*com*_ on visual tilt. However, it may also be explained by causal inference (Körding et al., 2007; Sato et al., 2007). Causal inference refers to a process where the brain judges whether or not multisensory signals share a common cause, and processes sensory signals differently depending on the outcome of this assessment. Put simply, signals that share a common cause will be combined, but signals that do not will be segregated; and signals deemed irrelevant may be discarded entirely. The assessment of causality is based on the degree of similarity between signals and an a-priori tolerance for discrepancies. Percepts will describe a mixture distribution of combinations and segregations, where combinations are most prominent for similar signals and segregations for dissimilar signals. In the context of spatial orientation and motion perception, evidence has been presented that this principle applies in heading perception (De Winkel et al., 2015, 2017; Acerbi et al., 2018); performing a Subjective Haptic Vertical task (De Winkel et al., 2018a); and possibly also when performing a Subjective Postural Vertical task (De Winkel and Nestmann, 2019; Nestmann et al., 2020). This principle can also account for observations of cue capture (Rock and Victor, 1964) in a spatial orientation task performed in microgravity and partial gravity, where the visual cue was discarded entirely (De Winkel et al., 2012). For the range of discrepancies tested in the present experiment, this strategy predicts that the average response will describe a sigmoid (Nestmann et al., 2020), similar to what was observed. Therefore, it is indistinguishable from the MLE model augmented with a heading dependency of visual variance, but it is considerably more complex. Particularities of causal inference may be distinguished from a tilt dependency in the variance of sensory signals by considering a larger range of discrepancies, because the influence of the visual signal on the average percept should reduce to zero in a more abrupt fashion for the causal inference model than for the MLE model (Nestmann et al., 2020). In addition, causal inference may become apparent by explicitly considering the shape of the response distribution over a large range of discrepancies. In light of these considerations, it is interesting to note that for one participant, the responses for $\varphi_{\text{AR}}=-36^{{}^{\circ}}$ on the RFT tend toward 0°, whereas an average bias of approximately 15° was predicted. It is possible that this participant (subconsciously) noticed the discrepancy and discarded the visual signal. However, because the present study only considered relatively small discrepancies, we consider a formal assessment beyond the scope of our work.

### Comparison With Previous Work

According to statistical models of perception, weights attributed to sensory signals should be inversely proportional to the variances of the sensory systems (Ernst and Banks, 2002; Hillis et al., 2002; Ernst and Bülthoff, 2004). It is unlikely that these variances themselves change depending on the task for which the information is used, yet, different weightings are observed between tasks. This is apparent when considering studies that derive weights by adopting a vector sum model (Mittelstaedt, 1983). We are aware of a number of studies that have investigated the perception of verticality via subjective visual vertical tasks similar to the RFT task. Specifically, Dyde et al. (2006) report relative weights for the body (i.e., the idiotropic vector), vision, and gravity (i.e., the vestibular and somatosensory systems) of 0.2:0.1:1.0; and average weights derived from Vingerhoets et al. (2009) are 0.23:0.11:1.0. This illustrates similar performance for a given task, despite differences in the experimental paradigm such as in the design of the visual stimulus, and using a different sample of the population. In contrast, when Dyde et al. (2006) used a task where the perceptual upright was derived from the interpretation of an ambiguous symbol “p” (or “d”), the weights changed drastically, to 2.6:1.2:1.0, despite using the same experimental conditions. We are not aware of similar evaluations of relative weights for the Subjective Postural Vertical, but note that Mittelstaedt (1983) already found that the idiotropic vector does not affect this task. These observations thus clearly illustrate that differences exist between tasks that exceed idiosyncratic variability.

These empirically observed weightings cannot be compared to those obtained using the present modeling directly, because what is represented by the components of the model is different: in the aforementioned studies, the body refers to the idiotropic vector, vision to the visual system, and gravity to the joint vestibular and somatosensory systems. In contrast, in the Clemens et al. (2011) study, somatosensory and vestibular contributions are separated by additionally considering how neck proprioceptors could be involved in the reference frame transformations. These are not considered in the vector sum model. In the present study, we additionally treat the vestibular and visual system as a joint head tilt sensor. In the Clemens et al. (2011) model and the present adaptation, the weights are attributed to partial representations of the body and head in space after reference frame transformations. These factors complicate an explicit comparison. Nevertheless, the modeling does show how different weightings can be obtained in a statistical framework despite fixed variances, namely by explicitly considering that different tasks probe internal representations that are constructed out of these signals differently.

A comparison can be made between the results of Clemens et al. and the present study for upright conditions (0° tilt). For this comparison, it is more convenient to compare estimates of the standard deviations for the different sensory systems directly, rather than the weights that can be derived from them. In their study, the average standard deviations for the head tilt sensors, body somatosensory system, and neck proprioceptors were 2.4, 10.8, and 4.9°, respectively. The idiotropic prior had an average value of 12.5°. In the present study, the idiotropic prior was omitted, but the values for the head, body, and neck sensors were 8.6, 0.8, and 9.3°. This means that here, the head tilt sensors and neck prioprioceptors were between two to four times *more* noisy, whereas the somatosensory system was ten times *less* noisy. The finding of increased noise in the head tilt signal is surprising, considering that the visual and vestibular system may be expected to produce redundant estimates of head tilt, which should theoretically reduce noise (Ernst and Banks, 2002; Hillis et al., 2002; Ernst and Bülthoff, 2004). A possible explanation for this increase in noise is that we used a variant of a reproduction paradigm, whereas Clemens et al. (2011) used a forced choice paradigm. The reproduction paradigm requires less trials and is more informative of the shape of the response distribution, but the data may be subject to additional sources of noise beyond what is purely perceptual. The large reduction of somatosensory noise may be due to the fact that participants were standing. This requires active maintenance of posture, which may provide continuous feedback on the somatosensory estimate of the vertical. In future work, standing and seated conditions may be compared directly.

### Conclusions

We observe a striking difference in the effect of visual stimuli between two experimental tasks designed to probe verticality perception. In the RFT task, we observe biases of ≈ 10° for visual tilts of 36°, vs. 0.25° in the SPV task. These observations can be explained if we assume that these tasks probe different internal representations of verticality, namely of the HIS and BIS. We show how these representations can be constructed by combining *direct* signals from head and body sensors, respectively, with *indirect* signals based on the alternative signal (i.e., body or head tilt, respectively) corrected for neck tilt. Due to this reference frame transformation, the weights for the direct and indirect representations are very different between these tasks. Perception of the BIS is dominated by body somatosensory signals, and this percept appears to be linked to posture maintenance. The HIS depends on head and body sensors about equally, provided that the sensors provide congruent information; when intersensory discrepancies are introduced, the weights shift in favor of the indirect signal. These results provide an explanation why different sensory weightings have been reported in studies that probe verticality perception in different ways, and also show that the body somatosensory system has a major impact spatial orientation.

## Data Availability Statement

The raw data supporting the conclusions of this article will be made available by the authors upon request, without undue reservation.

## Ethics Statement

The experiment was carried out in accordance with the declaration of Helsinki. All participants gave their written informed consent prior to participation. The experimental protocol was approved by the ethical committee of the medical faculty of the Eberhard-Karls University in Tübingen, Germany, under reference number 674/2018BO2.

## Author Contributions

KD: conceptualization, methodology, software, formal analysis, data curation, writing—original draft, writing—review and editing, visualization, supervision, and project administration. EE: methodology, investigation, writing—review and editing, and project administration. RH: writing—review and editing. HB: resources, writing—review and editing, and funding acquisition. All authors contributed to the article and approved the submitted version.

## Conflict of Interest

The authors declare that the research was conducted in the absence of any commercial or financial relationships that could be construed as a potential conflict of interest.

## Footnotes

^1^Averages for each condition can be calculated using the LME models and coefficients. The peak-to-peak difference can be calculated using the intercept, 8.357°, which corresponds to the average at $\varphi_{\text{AR}}=-36^{{}^{\circ}}$, and the coefficient for $\varphi_{\text{AR}}=36^{{}^{\circ}}$ (−18.719°), which gives an average of 8.357 + (−18.719) = −10.362° for φ = 36°. The average maximum bias is [8.357 − (−10.362)]/2 = 9.360.

## Abbreviations

AR: alternative reality
BIC: Bayesian information criterion
BIS: body in space
COP: center of pressure
HIS: head in space
HOB: head on body
LME: linear mixed effects
MAP: maximum A-posteriori
MLE: maximum likelihood estimation
OCR: ocular counter-roll
RFT: rod and frame test
SPV: subjective postural vertical.

## Appendix

### List of Symbols

**Table:**

ω_*k*_	weights for direct (*k* = HISd, BISd) and indirect (*k* = {HISi, BISi}) representations of the Head and Body In Space
φ_*m*_	roll tilt angle of stimulus modality *m*, with *m* = {AR, N, P, V} for the AR system, neck, platform and vision, respectively.
μ_*n*_	mean signal for sensory system *n*, with *n* = {*vis, ves, pro, som*} for the visual, vestibular, proprioceptive and somatosensory systems, and com for a combined visual-vestibular head tilt signal.
σ_*n*_	standard deviation for sensory system *n*
*K*_*n*_	gain of tilt-dependency in standard deviation sensory system *n*
*r*_*j*_	responses on the Rod & Frame Test (*j* = RFT) and Subjective Postural Vertical (*j* = SPV) tasks
*x*_*n*_	signal from sensory system *n*.

## References

[B1] Acerbi L. Dokka K. Angelaki D. E. Ma W. J. (2018). Bayesian comparison of explicit and implicit causal inference strategies in multisensory heading perception. PLoS Comput. Biol. 14:e1006110 10.1371/journal.pcbi.1006110 30052625 PMC6063401

[B2] Alberts B. B. de Brouwer A. J. Selen L. P. Medendorp W. P. (2016). A bayesian account of visual-vestibular interactions in the rod-and-frame task. eneuro 3. 10.1523/ENEURO.0093-16.2016 PMC5093328 27844055

[B3] Anastasopoulos D. Haslwanter T. Bronstein A. Fetter M. Dichgans J. (1997). Dissociation between the perception of body verticality and the visual vertical in acute peripheral vestibular disorder in humans. Neurosci. Lett. 233, 151–153. 10.1016/S0304-3940(97)00639-3 9350855

[B4] Angelaki D. E. Cullen K. E. (2008). Vestibular system: the many facets of a multimodal sense. Annu. Rev. Neurosci. 31, 125–150. 10.1146/annurev.neuro.31.060407.125555 18338968

[B5] Barnett-Cowan M. Dyde R. T. Harris L. R. (2005). Is an internal model of head orientation necessary for oculomotor control? Ann. N. Y. Acad. Sci. 1039, 314–324. 10.1196/annals.1325.030 15826985

[B6] Barnett-Cowan M. Fleming R. W. Singh M. Bülthoff H. H. (2011). Perceived object stability depends on multisensory estimates of gravity. PLoS ONE 6:e19289. 10.1371/journal.pone.0019289 21556363 PMC3083421

[B7] Barnett-Cowan M. Harris L. R. (2008). Perceived self-orientation in allocentric and egocentric space: effects of visual and physical tilt on saccadic and tactile measures. Brain Res. 1242, 231–243. 10.1016/j.brainres.2008.07.075 18706895

[B8] Barnett-Cowan M. Jenkin H. L. Dyde R. T. Jenkin M. R. Harris L. R. (2013). Asymmetrical representation of body orientation. J. Vis. 13:3. 10.1167/13.2.3 23378132

[B9] Bisdorff A. Wolsley C. Anastasopoulos D. Bronstein A. Gresty M. (1996). The perception of body verticality (subjective postural vertical) in peripheral and central vestibular disorders. Brain 119, 1523–1534. 10.1093/brain/119.5.1523 8931577

[B10] Bortolami S. B. Pierobon A. DiZio P. Lackner J. R. (2006). Localization of the subjective vertical during roll, pitch, and recumbent yaw body tilt. Exp. Brain Res. 173, 364–373. 10.1007/s00221-006-0385-y 16628401

[B11] Brecher G. A. (1934). Die optokinetische auslösung von augenrollung und rotatorischem nystagmus. Pflüger's Arch. Gesamte Physiol. Menschen und der Tiere 234, 13–28. 10.1007/BF01766880

[B12] Bronstein A. M. (1986). Suppression of visually evoked postural responses. Exp. Brain Res. 63, 655–658. 10.1007/BF00237488 3489640

[B13] Carver S. Kiemel T. Van Der Kooij H. Jeka J. J. (2005). Comparing internal models of the dynamics of the visual environment. Biol. Cybern. 92, 147–163. 10.1007/s00422-004-0535-x 15703940

[B14] Cheung B. Howard I. (1991). Optokinetic torsion: dynamics and relation to circularvection. Vis. Res. 31, 1327–1335. 10.1016/0042-6989(91)90054-9 1891821

[B15] Cheung B. Money K. Howard I. Kirienko N. Johnson W. Lackner J.. (1992). Human ocular torsion during parabolic flights: an analysis with scleral search coil. Exp. Brain Res. 90, 180–188. 10.1007/BF00229270 1521606

[B16] Cian C. Esquivié D. Barraud P. A. Raphel C. (1995). Respective contribution of orientation contrast and illusion of self-tilt to the rod-and-frame effect. Perception 24, 623–630. 10.1068/p240623 7478903

[B17] Clark B. Graybiel A. (1963). Perception of the postural vertical in normals and subjects with labyrinthine defects. J. Exp. Psychol. 65:490. 10.1037/h0045606 14021495

[B18] Clemens I. A. De Vrijer M. Selen L. P. Van Gisbergen J. A. Medendorp W. P. (2011). Multisensory processing in spatial orientation: an inverse probabilistic approach. J. Neurosci. 31, 5365–5377. 10.1523/JNEUROSCI.6472-10.2011 21471371 PMC6622694

[B19] De Winkel K. N. Clément G. Groen E. L. Werkhoven P. J. (2012). The perception of verticality in lunar and martian gravity conditions. Neurosci. Lett. 529, 7–11. 10.1016/j.neulet.2012.09.026 22999922

[B20] De Winkel K. N. Katliar M. Bülthoff H. H. (2015). Forced fusion in multisensory heading estimation. PLoS ONE 10:e0127104. 10.1371/journal.pone.0127104 25938235 PMC4418840

[B21] De Winkel K. N. Katliar M. Bülthoff H. H. (2017). Causal inference in multisensory heading estimation. PLoS ONE 12:e0169676. 10.1371/journal.pone.0169676 28060957 PMC5218471

[B22] De Winkel K. N. Katliar M. Diers D. Bülthoff H. H. (2018a). Causal inference in the perception of verticality. Sci. Rep. 8:5483. 10.1038/s41598-018-23838-w 29615728 PMC5882842

[B23] De Winkel K. N. Kurtz M. Bülthoff H. H. (2018b). Effects of visual stimulus characteristics and individual differences in heading estimation. J. Vis. 18:9. 10.1167/18.11.9 30347100

[B24] De Winkel K. N. Nestmann S. (2019). “Increased susceptibility to visually induced biases in verticality perception with age,” in Neurologie & Rehabilitation, Vol. S1, Human Perception of Verticality: Lateropulsion & Retropulsion in Neurological Disorders, eds B. Bülau and R. Engels (Bad Honnef: Hippocampus Verlag e.K), S62.

[B25] Dijkstra T. M. H. Schöner G. Giese M. A. Gielen C. C. A. M. (1994). Frequency dependence of the action-perception cycle for postural control in a moving visual environment: relative phase dynamics. Biol. Cybern. 71, 489–501. 10.1007/BF00198467 7999875

[B26] Dyde R. T. Jenkin M. R. Harris L. R. (2006). The subjective visual vertical and the perceptual upright. Exp. Brain Res. 173, 612–622. 10.1007/s00221-006-0405-y 16550392

[B27] Eggert T. (1998). Der Einfluss orientierter Texturen auf die subjektive Vertikale und seine systemtheoretische analyse (Ph.D. thesis). Technical University of Munich, Munich, Germany.

[B28] Ernst M. O. Banks M. S. (2002). Humans integrate visual and haptic information in a statistically optimal fashion. Nature 415:429. 10.1038/415429a 11807554

[B29] Ernst M. O. Bülthoff H. H. (2004). Merging the senses into a robust percept. Trends Cogn. Sci. 8, 162–169. 10.1016/j.tics.2004.02.002 15050512

[B30] Farooq S. Proudlock F. Gottlob I. (2004). Torsional optokinetic nystagmus: normal response characteristics. Br. J. Ophthalmol. 88, 796–802. 10.1136/bjo.2003.028738 15148215 PMC1772190

[B31] Fraser L. E. Makooie B. Harris L. R. (2015). The subjective visual vertical and the subjective haptic vertical access different gravity estimates. PLoS ONE 10:e0145528. 10.1371/journal.pone.0145528 26716835 PMC4696803

[B32] Golomer E. Crémieux J. Dupui P. Isableu B. Ohlmann T. (1999). Visual contribution to self-induced body sway frequencies and visual perception of male professional dancers. Neurosci. Lett. 267, 189–192. 10.1016/S0304-3940(99)00356-0 10381008

[B33] Happee R. de Bruijn E. Forbes P. A. van der Helm F. C. (2017). Dynamic head-neck stabilization and modulation with perturbation bandwidth investigated using a multisegment neuromuscular model. J. Biomech. 58, 203–211. 10.1016/j.jbiomech.2017.05.005 28577906

[B34] Harris L. R. Jenkin M. Dyde R. T. Jenkin H. (2011). “Enhancing visual cues to orientation: Suggestions for space travelers and the elderly,” in Progress in Brain Research, Vol. 191, eds A. M. Green, C. E. Chapman, J. F. Kalaska, and F. Lepore (Amsterdam: Elsevier), 133–142. 10.1016/B978-0-444-53752-2.00008-4 21741549

[B35] Hillis J. M. Ernst M. O. Banks M. S. Landy M. S. (2002). Combining sensory information: mandatory fusion within, but not between, senses. Science 298, 1627–1630. 10.1126/science.1075396 12446912

[B36] Howard I. P. (1982). Human Visual Orientation. Chichester; Sussex; New York, NY: John Wiley and Sons.

[B37] Howard I. P. Bergström S. S. Ohmi M. (1990). Shape from shading in different frames of reference. Perception 19, 523–530. 10.1068/p190523 2096370

[B38] Isableu B. Ohlmann T. Crémieux J. Amblard B. (1997). Selection of spatial frame of reference and postural control variability. Exp. Brain Res. 114, 584–589. 10.1007/PL00005667 9187294

[B39] Karnath H.-O. Ferber S. Dichgans J. (2000). The origin of contraversive pushing: evidence for a second graviceptive system in humans. Neurology 55, 1298–1304. 10.1212/WNL.55.9.1298 11087771

[B40] Karnath H.-O. Johannsen L. Broetz D. Küker W. (2005). Posterior thalamic hemorrhage induces “pusher syndrome”. Neurology 64, 1014–1019. 10.1212/01.WNL.0000154527.72841.4A 15781819

[B41] Kass R. E. Raftery A. E (1995). Bayes factors. J. Am. Stat. Assoc. 90, 773–795. 10.1080/01621459.1995.10476572

[B42] Kheradmand A. Winnick A. (2017). Perception of upright: multisensory convergence and the role of temporo-parietal cortex. Front. Neurol. 8:552. 10.3389/fneur.2017.00552 29118736 PMC5660972

[B43] Kingma H. Stegeman P. Vogels R. (1997). Ocular torsion induced by static and dynamic visual stimulation and static whole body roll. Eur. Arch. Oto-rhino-laryngol. 254, S61–S63. 10.1007/BF02439726 9065630

[B44] Körding K. P. Beierholm U. Ma W. J. Quartz S. Tenenbaum J. B. Shams L. (2007). Causal inference in multisensory perception. PLoS ONE 2:e943. 10.1371/journal.pone.0000943 17895984 PMC1978520

[B45] Lee D. N. Aronson E. (1974). Visual proprioceptive control of standing in human infants. Percept. Psychophys. 15, 529–532. 10.3758/BF0319929716338048

[B46] Lee D. N. Lishman J. R. (1975). Visual proprioceptive control of stance. J. Hum. Mov. Stud. 1, 87–95.

[B47] Lestienne F. Soechting J. Berthoz A. (1977). Postural readjustments induced by linear motion of visual scenes. Exp. Brain Res. 28, 363–384. 10.1007/BF00235717 885185

[B48] Li L. Rehr R. Bruns P. Gerkmann T. Röder B. (2020). A survey on probabilistic models in human perception and machines. Front. Robot. AI 7:85 10.3389/frobt.2020.00085 PMC7805657 33501252

[B49] Loomis A. (2011). Figure Drawing for All It's Worth. London: Titan books ltd.

[B50] Mann C. W. Berthelot-Berry N. H. Dauterive H. J. Jr. (1949). The perception of the vertical: I. visual and non-labyrinthine cues. J. Exp. Psychol. 39:538. 10.1037/h0063533 18140137

[B51] Medendorp W. P. Alberts B. B. Verhagen W. I. Koppen M. Selen L. P. (2018). Psychophysical evaluation of sensory reweighting in bilateral vestibulopathy. Front. Neurol. 9:377. 10.3389/fneur.2018.00377 29910766 PMC5992424

[B52] Miller E. F. (1962). Counterrolling of the human eyes produced by head tilt with respect to gravity. Acta Oto-laryngol. 54, 479–501. 10.3109/00016486209126967 14473991

[B53] Mittelstaedt H. (1983). A new solution to the problem of the subjective vertical. Naturwissenschaften 70, 272–281. 10.1007/BF00404833 6877388

[B54] Mittelstaedt H. (1995). “The formation of the visual and the postural vertical,” in Multisensory control of Posture, eds F. Hlavacka and T. Mergner (New York, NY: Springer), 147–155. 10.1007/978-1-4615-1931-7_18

[B55] Mittelstaedt H. (1996). Somatic graviception. Biol. Psychol. 42, 53–74. 10.1016/0301-0511(95)05146-5 8770370

[B56] Mittelstaedt H. (1998). Origin and processing of postural information. Neurosci. Biobehav. Rev. 22, 473–478. 10.1016/S0149-7634(97)00032-8 9595557

[B57] Nestmann S. Karnath H.-O. Bülthoff H. H. De Winkel K. N. (2020). Changes in the perception of upright body orientation with age. PLoS ONE 15:e0233160. 10.1371/journal.pone.0233160 32469902 PMC7259641

[B58] Oie K. S. Kiemel T. Jeka J. J. (2002). Multisensory fusion: simultaneous re-weighting of vision and touch for the control of human posture. Cogn. Brain Res. 14, 164–176. 10.1016/S0926-6410(02)00071-X 12063140

[B59] Oman C. M. (2003). “Human visual orientation in weightlessness,” in Levels of Perception, eds L. R. Harris and M. Jenkin (New York, NY: Springer), 375–398. 10.1007/0-387-22673-7_19

[B60] Perennou D. Amblard B. Leblond C. Pelissier J. (1998). Biased postural vertical in humans with hemispheric cerebral lesions. Neurosci. Lett. 252, 75–78. 10.1016/S0304-3940(98)00501-1 9756325

[B61] Peterka R. J. (2002). Sensorimotor integration in human postural control. J. Neurophysiol. 88, 1097–1118. 10.1152/jn.2002.88.3.1097 12205132

[B62] Rock I. Victor J. (1964). Vision and touch: an experimentally created conflict between the two senses. Science 143, 594–596. 10.1126/science.143.3606.594 14080333

[B63] Sato Y. Toyoizumi T. Aihara K. (2007). Bayesian inference explains perception of unity and ventriloquism aftereffect: identification of common sources of audiovisual stimuli. Neural Comput. 19, 3335–3355. 10.1162/neco.2007.19.12.3335 17970656

[B64] Schwarz G. (1978). Estimating the dimension of a model. Ann. Stat. 6, 461–464. 10.1214/aos/1176344136

[B65] Vaitl D. Mittelstaedt H. Saborowski R. Stark R. Baisch F. (2002). Shifts in blood volume alter the perception of posture: further evidence for somatic graviception. Int. J. Psychophysiol. 44, 1–11. 10.1016/S0167-8760(01)00184-2 11852154

[B66] Van Asten W. N. J. C. Gielen C. C. A. M. Van Der Gon J. J. D. (1988). Postural adjustments induced by simulated motion of differently structured environments. Exp. Brain Res. 73, 371–383. 10.1007/BF00248230 3215313

[B67] Van der Kooij H. Jacobs R. Koopman B. Grootenboer H. (1999). A multisensory integration model of human stance control. Biol. Cybern. 80, 299–308. 10.1007/s004220050527 10365423

[B68] Van der Kooij H. Jacobs R. Koopman B. Van der Helm F. (2001). An adaptive model of sensory integration in a dynamic environment applied to human stance control. Biol. Cybern. 84, 103–115. 10.1007/s004220000196 11205347

[B69] Vingerhoets R. A. A. De Vrijer M. Van Gisbergen J. A. Medendorp W. P. (2009). Fusion of visual and vestibular tilt cues in the perception of visual vertical. J. Neurophysiol. 101, 1321–1333. 10.1152/jn.90725.2008 19118112

[B70] Wade S. W. Curthoys I. S. (1997). The effect of ocular torsional position on perception of the roll-tilt of visual stimuli. Vis. Res. 37, 1071–1078. 10.1016/S0042-6989(96)00252-0 9196725

[B71] Wenderoth P. (1974). The distinction between the rod-and-frame illusion and the rod-and-frame test. Perception 3, 205–212. 10.1068/p030205 4457823

[B72] Wilkinson G. N. Rogers C. E. (1973). Symbolic description of factorial models for analysis of variance. J. R. Stat. Soc. Ser. C 22, 392–399. 10.2307/2346786

[B73] Witkin H. A. Asch S. E. (1948). Studies in space orientation. IV. Further experiments on perception of the upright with displaced visual fields. J. Exp. Psychol. 38:762 10.1037/h0053671 18893191

[B74] Yuille A. Bülthoff H. (1996). “Bayesian decision theory and psychophysics,” in Perception as Bayesian Inference, eds W. Richards and D. C. Knill (Cambridge: Cambridge University Press), 123–161. 10.1017/CBO9780511984037.006

[B75] Zaichik L. Rodchenko V. Rufov I. Yashin Y. White A. (1999). “Acceleration perception,” in Modeling and Simulation Technologies Conference and Exhibit (Reston, VA), 4334 10.2514/6.1999-4334

