# Supplementary material for: Multisensory Interactions in Head and Body Centered Perception of Verticality
Source: Front Neurosci. 2021 Jan 12;14:599226. doi: 10.3389/fnins.2020.599226 (PMC7835726; doi:10.3389/fnins.2020.599226)

Max Planck Institute for Biological Cybernetics

Department of Perception, Cognition and Action

Max-Planck-Ring 14 ◆ D-72076 ◆ Tübingen

Interactions between the Subjective Visual and Postural representations of Verticality

Instructions for participants

Dear sir/madam,

Thank you for your interest in participating in this study. The purpose of this text is to provide you with information regarding the goals and design of the experiment 'Interactions between the Subjective Visual and Postural representations of Verticality'. This information should allow you to make a well-informed decision regarding your willingness to participate in this study.

The present study is designed to determine how our brain constructs percepts of upright, how these percepts affect balance, and how this process may change as we age. Resolving this question is of importance to neuroscience and may ultimately have applications in rehabilitation after stroke.
It is known that visual and physical information contribute to perception of an upright. To determine exactly how this information is combined, we will manipulate what you see and feel as upright while you stand on a motion platform. These manipulations will be achieved by using a Head-Mounted Display (HMD) and a motion platform, on a series of experimental *trials*. On each trial, the platform and HMD will independently tilt to particular target angles. It is your task to estimate the true direction of upright (that is, gravity) in two different tasks:

- Subjective Visual Vertical: a rod is shown in the image visible in the HMD. Your task is to adjust the orientation of the rod until it aligns with your feeling of upright using a game controller. Imagine that the rod reflects your body and put it upright to the real world (in other words: such that it aligns with the direction of gravity).
- Subjective Postural Vertical: when the platform and HMD have reached the target tilt angles, use the game controller to adjust the platform orientation until the platform is level, and you are upright.

Both tasks will be performed with the head aligned with the body, as well as with the head tilted to the right, making a total of four experimental blocks.

Try to perform the tasks intuitively: when the motions have stopped, orient the rod (SVV) or platform (SPV) to align with your first impression. Do not close your eyes or sway your body intentionally. Focus your gaze on the pink post-it note that is attached to the middle of the hand rail on the stairs, and do not look down to your feet. Confirm your responses and advance to new trials by button press. The experimenter will show you which buttons to use to perform each task.

Dr. K.N. de Winkel
Max Planck Institute for Biological Cybernetics
ksander.dewinkel@tuebingen.mpg.de

For each block with the head upright, there will be 63 trials; for each block with the head tilted, there will be 30 trials. After each block there will be a break. The experimenter will tell you the conditions of a block before you start.
Before the experiment begins there will be a small number of training trials to get used to the simulator and the tasks.

Including instructions, training trials, and (compulsory) breaks, each session will take approximately two to three hours to complete. You will be compensated for your time at a rate of € 8, - per hour. We aim to collect data from 20 participants; one group of younger and one group of older participants.

There are no known (dis-)advantages for you personally as a result of participating in this study, although there is a risk of motion sickness.

Motion sickness can be recognized from the following symptoms:

- General discomfort
- Fatigue
- Headache
- Eyestrain
- Difficulty focusing (visually)
- Increased salivation
- Sweating
- Nausea
- Difficulty concentrating
- Fullness of the head
- Blurred vision
- Dizzy[1] (eyes open)
- Dizzy[1] (eyes closed)
- Vertigo[2]
- Stomach awareness

[1]Dizziness is a feeling of light-headedness or equilibrium imbalance
[2]Vertigo is the feeling that either you or the surroundings are spinning

If you experience symptoms of motion sickness at any point in the experiment or if at any time you wish to have an extra break, then please do not hesitate to communicate this immediately to the researcher. The experiment will then be paused or stopped.

Note that your participation is entirely voluntary and you may withdraw from the study at any time without penalty.

Dr. K.N. de Winkel
Max Planck Institute for Biological Cybernetics
ksander.dewinkel@tuebingen.mpg.de

Multisensory interactions in head and body centered perception of verticality

De Winkel, Ksander N. K.N.deWinkel@tudelft.nl

Edel, Ellen ellened70@outlook.de

Happee, Riender R.Happee@tudelft.nl

Bülthoff, Heinrich H. heinrich.buelthoff@tuebingen.mpg.de

Supplementary material – Each figure shows the data and model fit for an individual subject, specified by the 'Participant id' noted above the figure. For each panel, the row specifies the task (RFT – Rod & Frame Test task, SPV – Subjective Postural Vertical task); the column specifies neck tilt (head up, head tilted). Data colored in blue, orange, and yellow represent observations for platform tilts φ_P of -3°, 0°, 3°, respectively. Individual observations are represented by semi-transparent points. The x-axis specifies the AR system tilt angle, the y-axis the response tilt angle. The lines represent the predicted mean response, with colors matching the φ_P condition. The shaded areas represent ±1 standard deviation predicted for φ_P =0° conditions.

Participant id 1

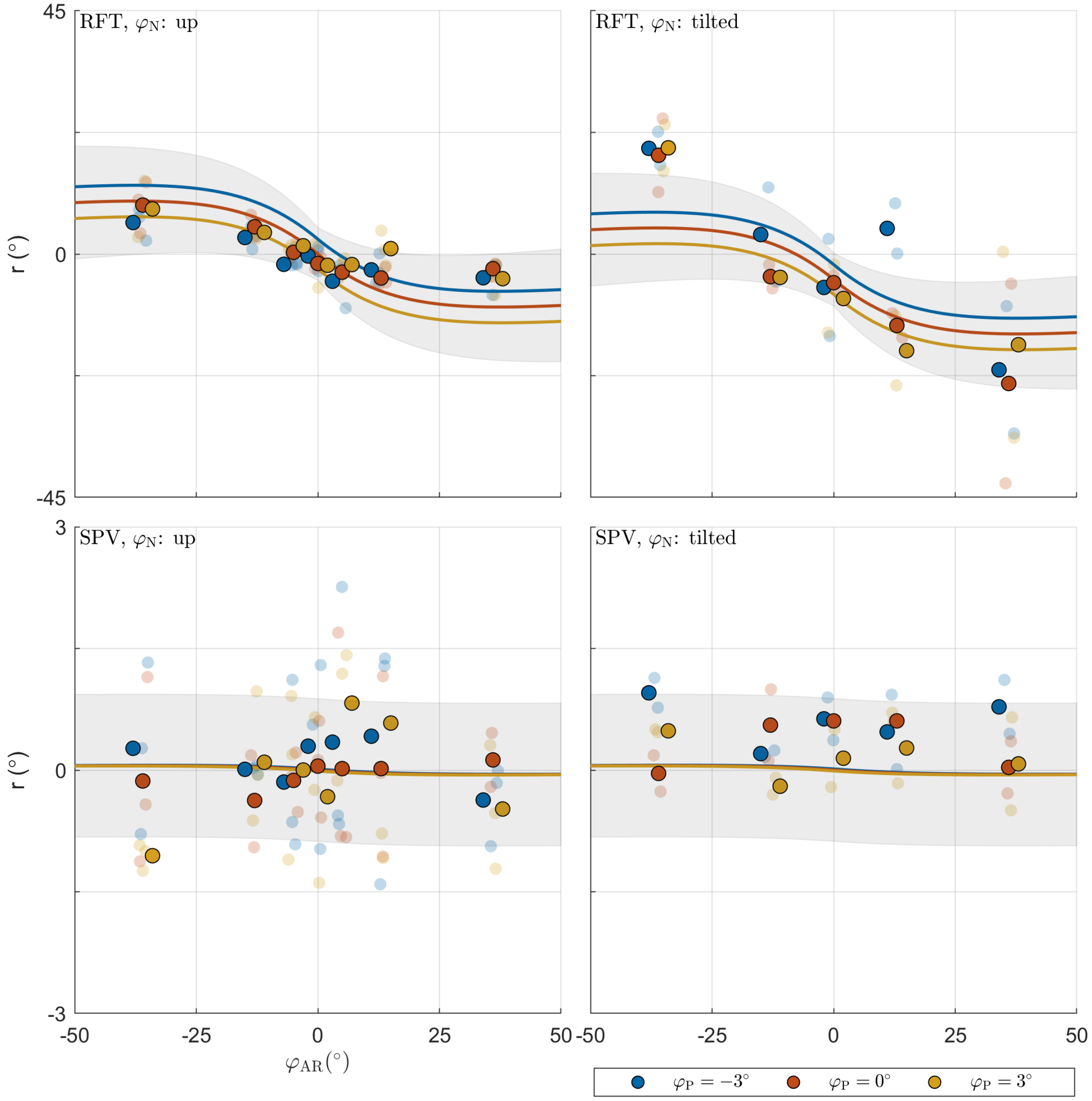

Participant id 2

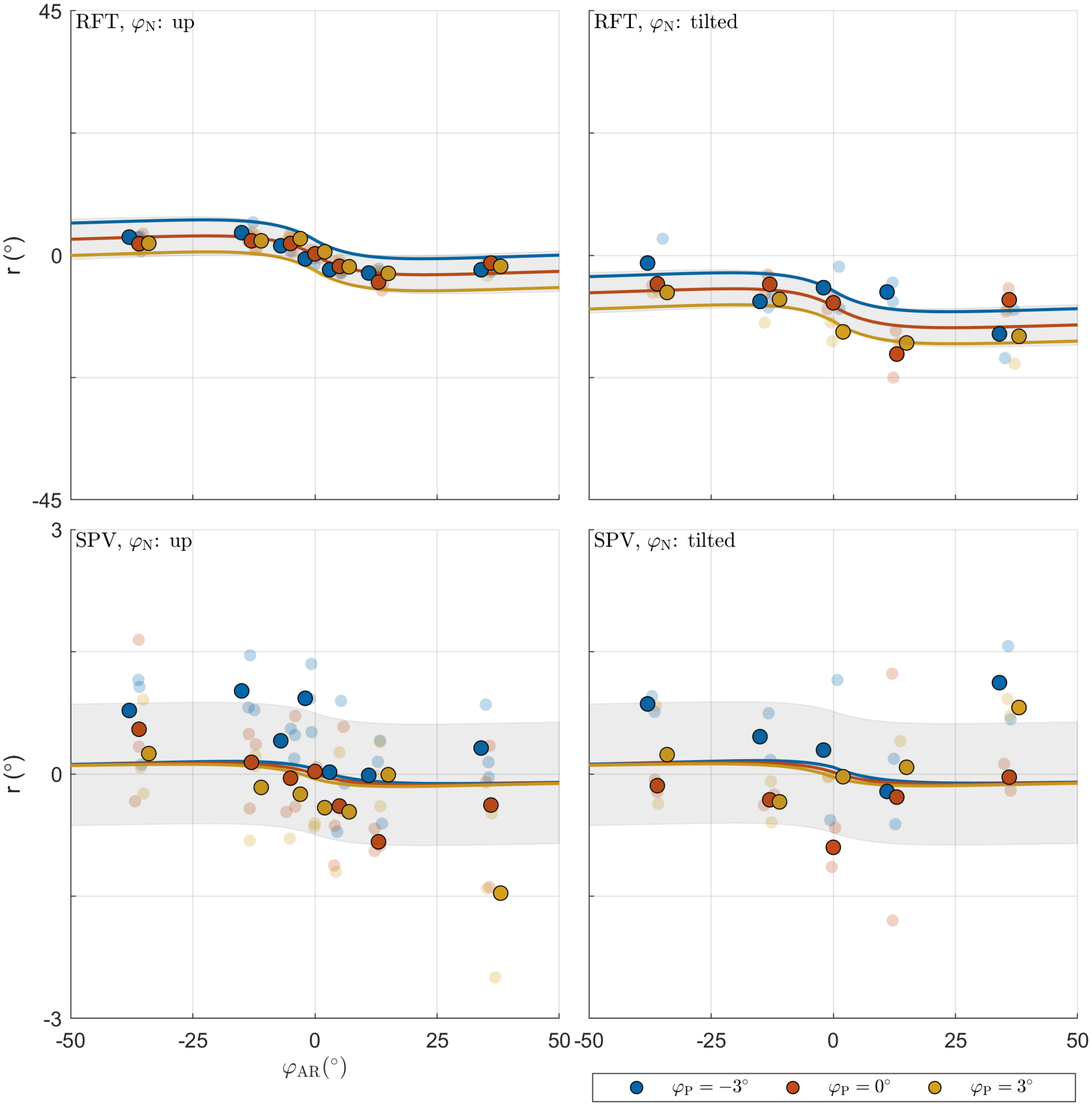

Participant id 3

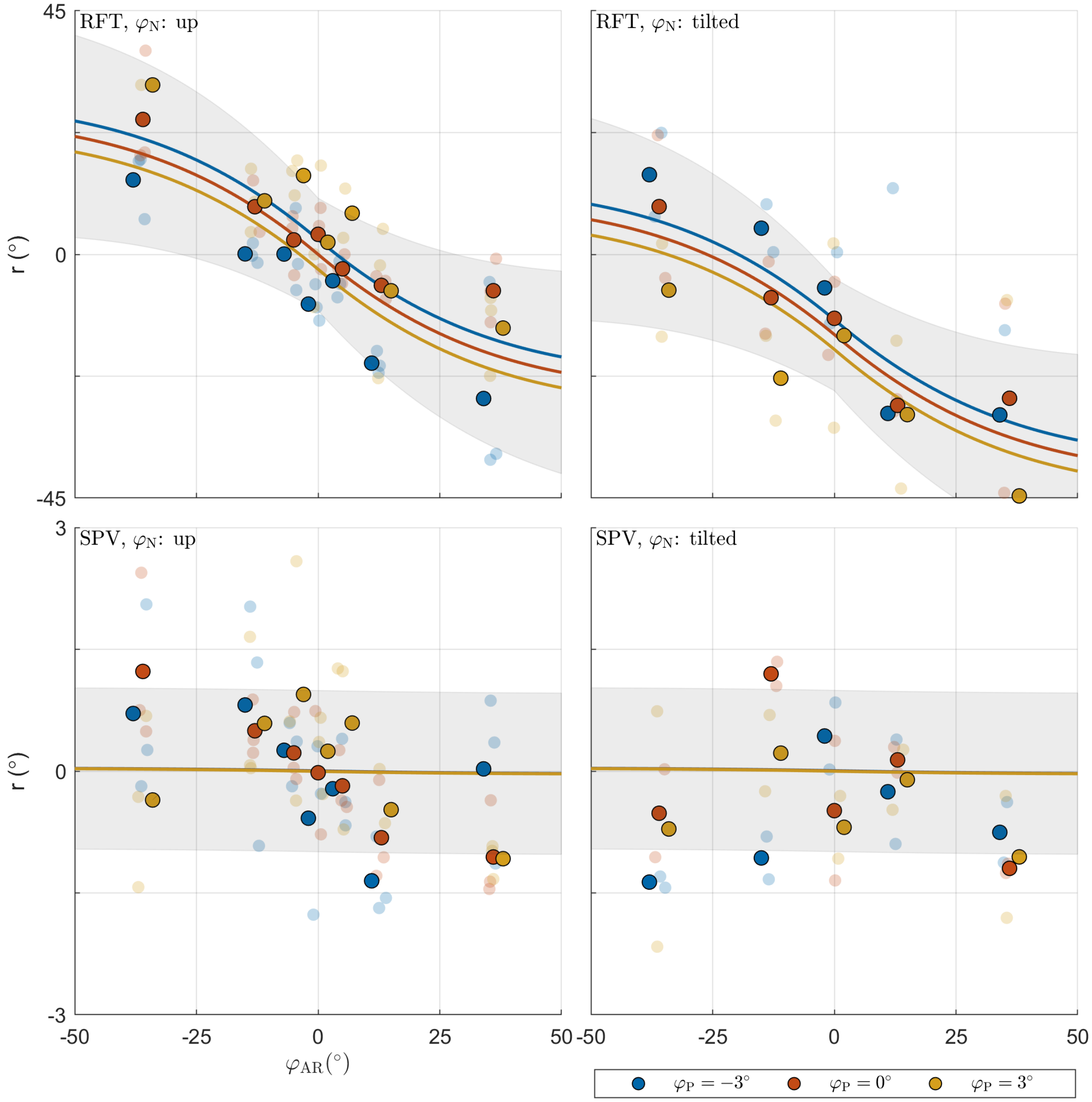

Participant id 4 (additional negative gain on somatosensory percept; see main text)

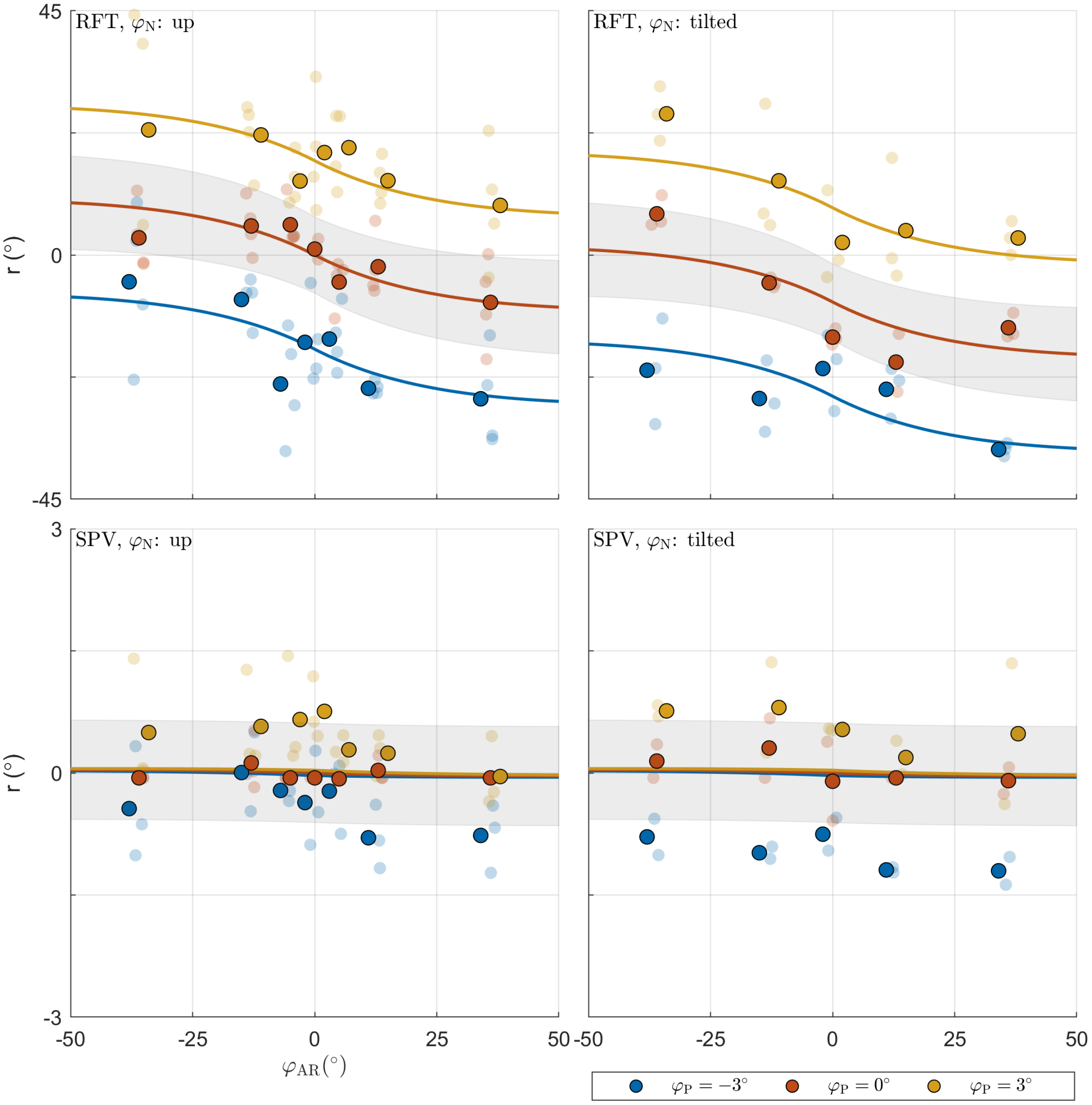

Participant id 5

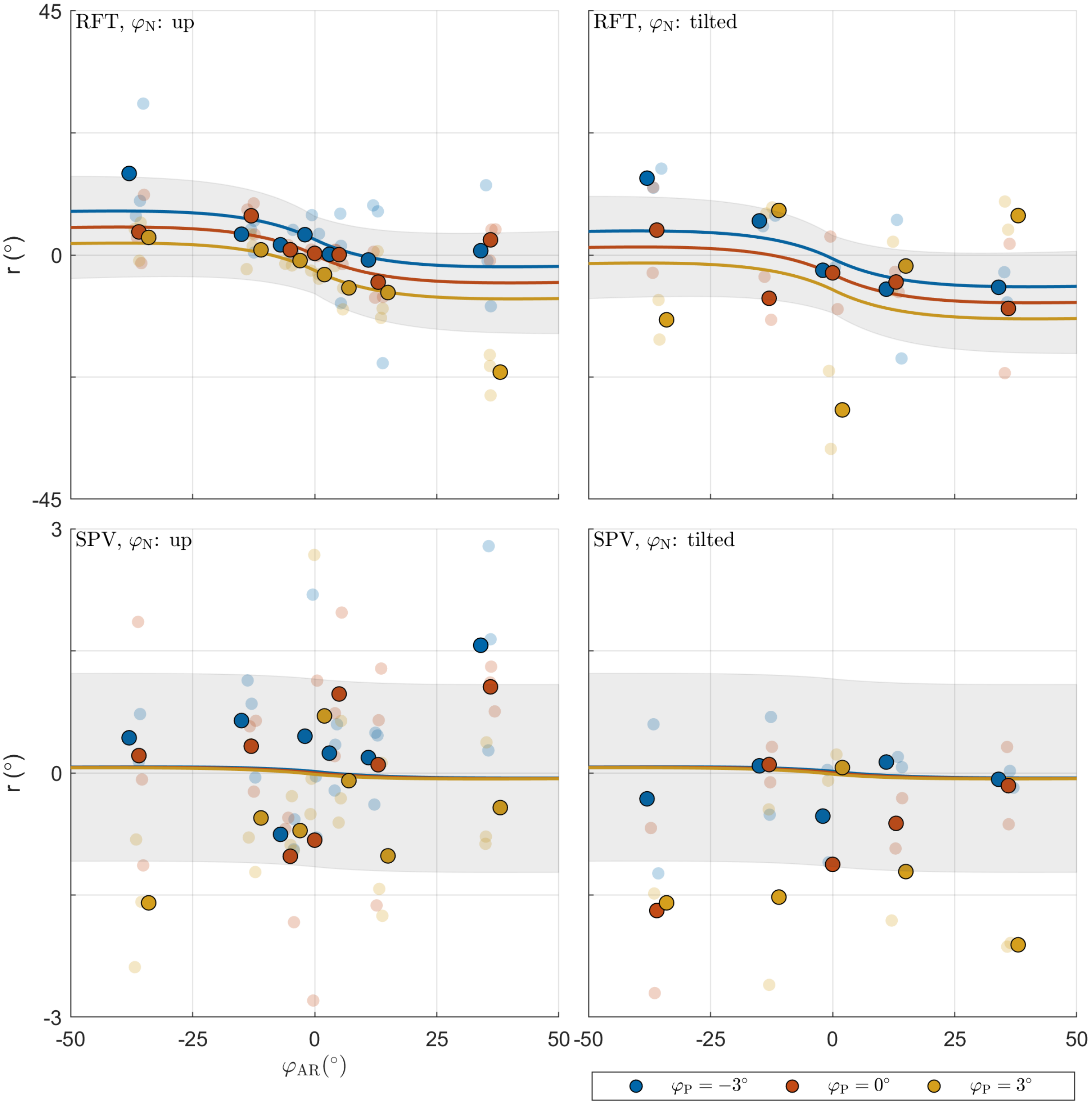

Participant id 6

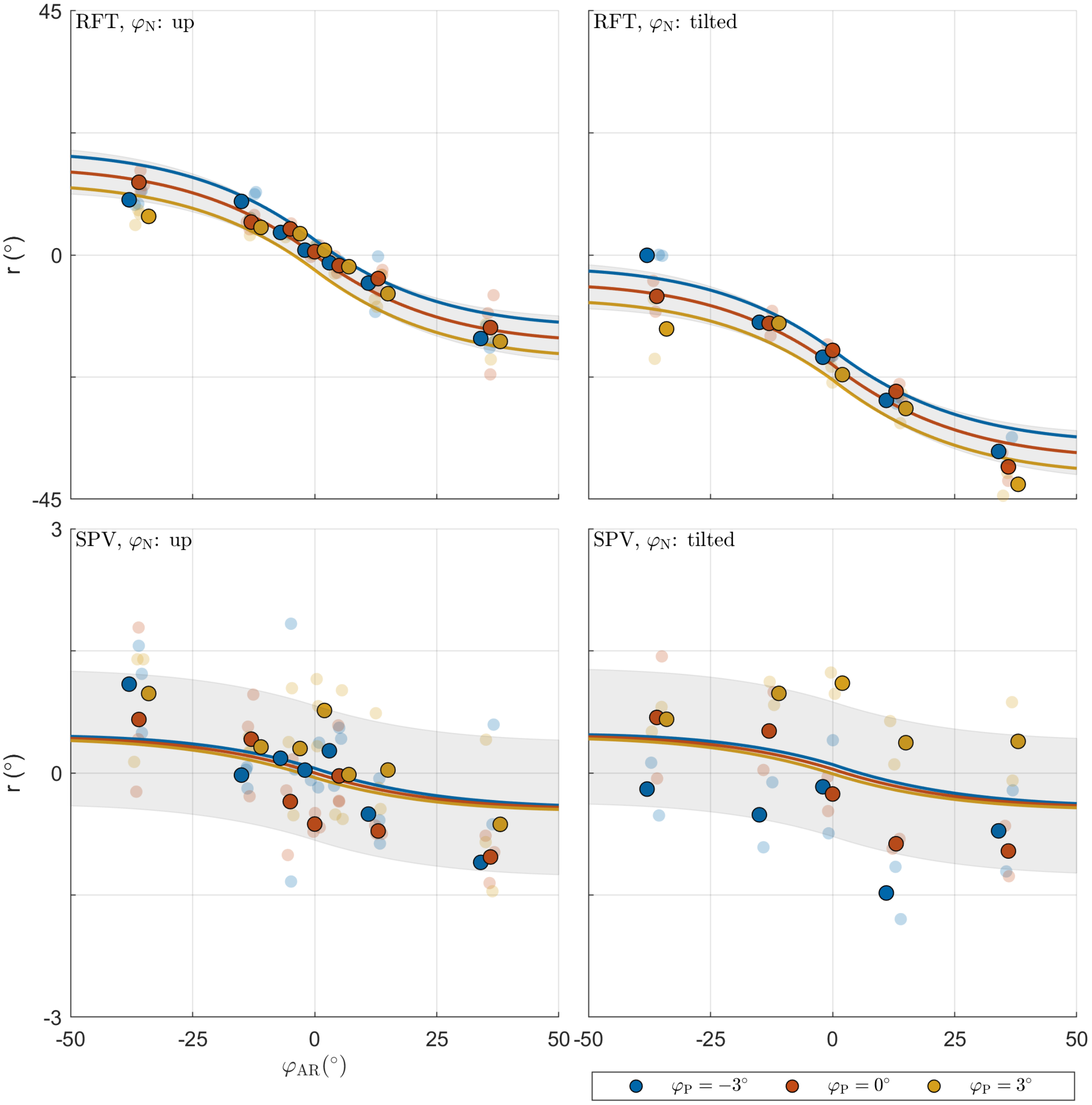

Participant id 7

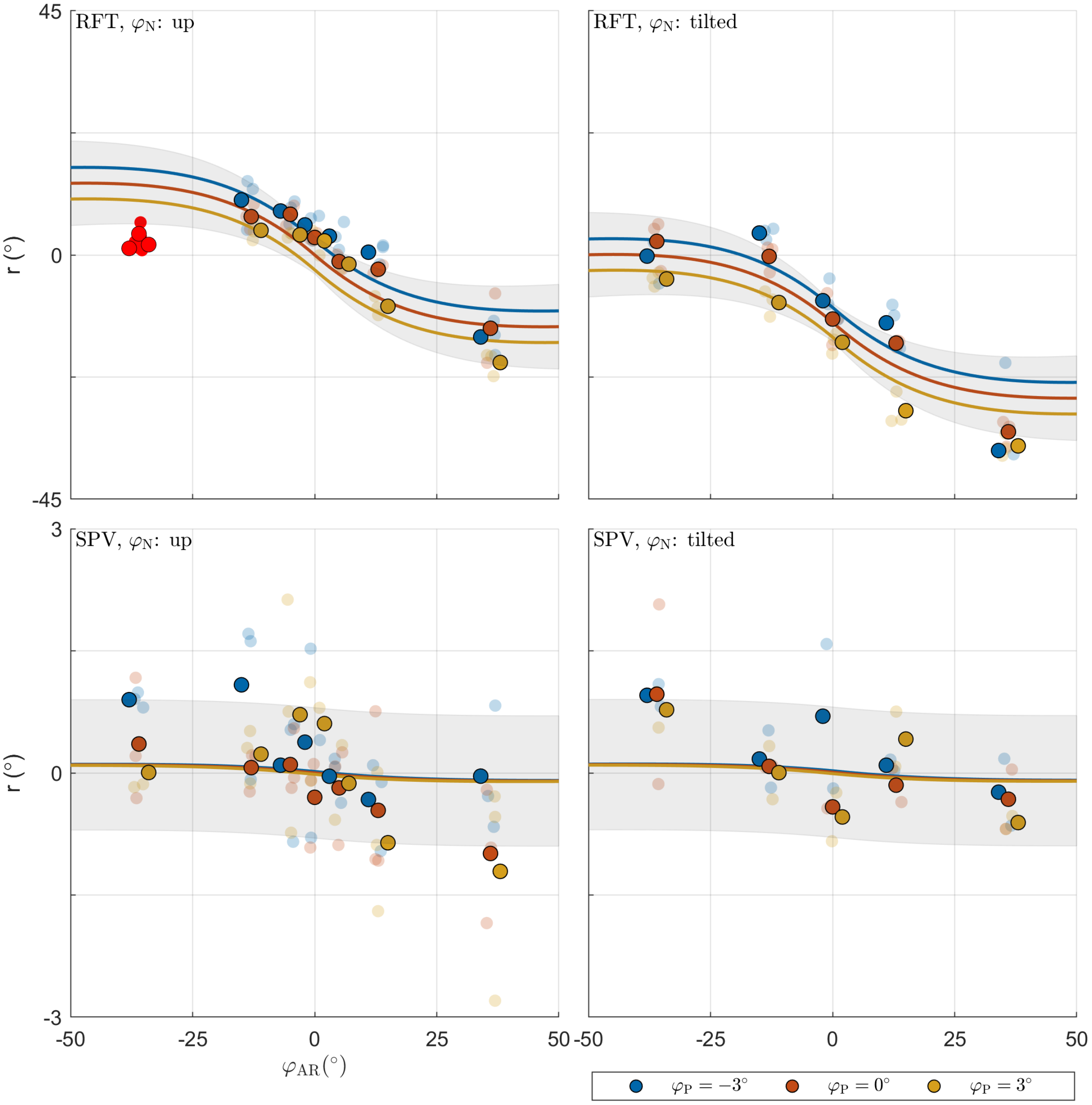

Participant id 8

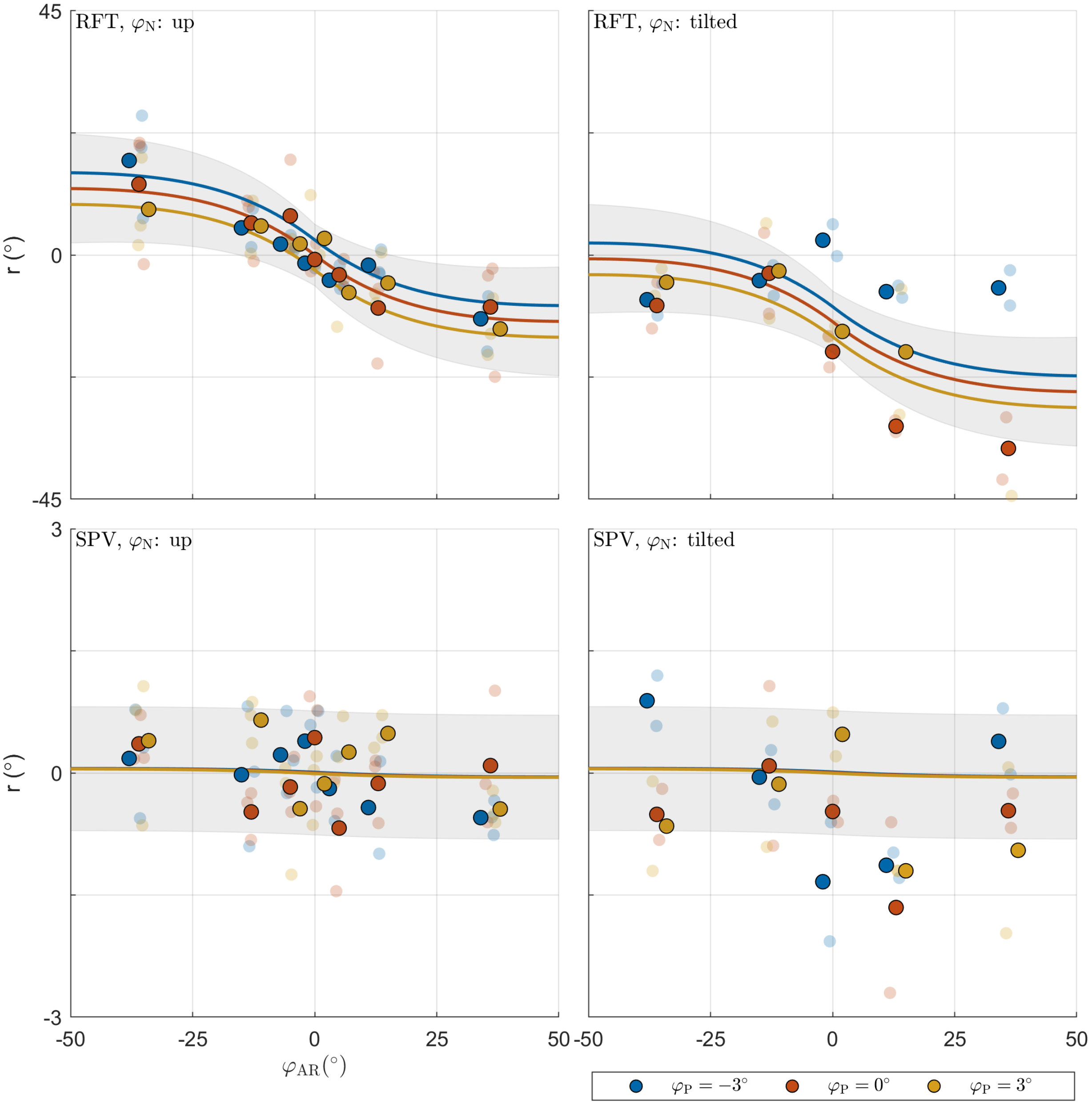

Participant id 9

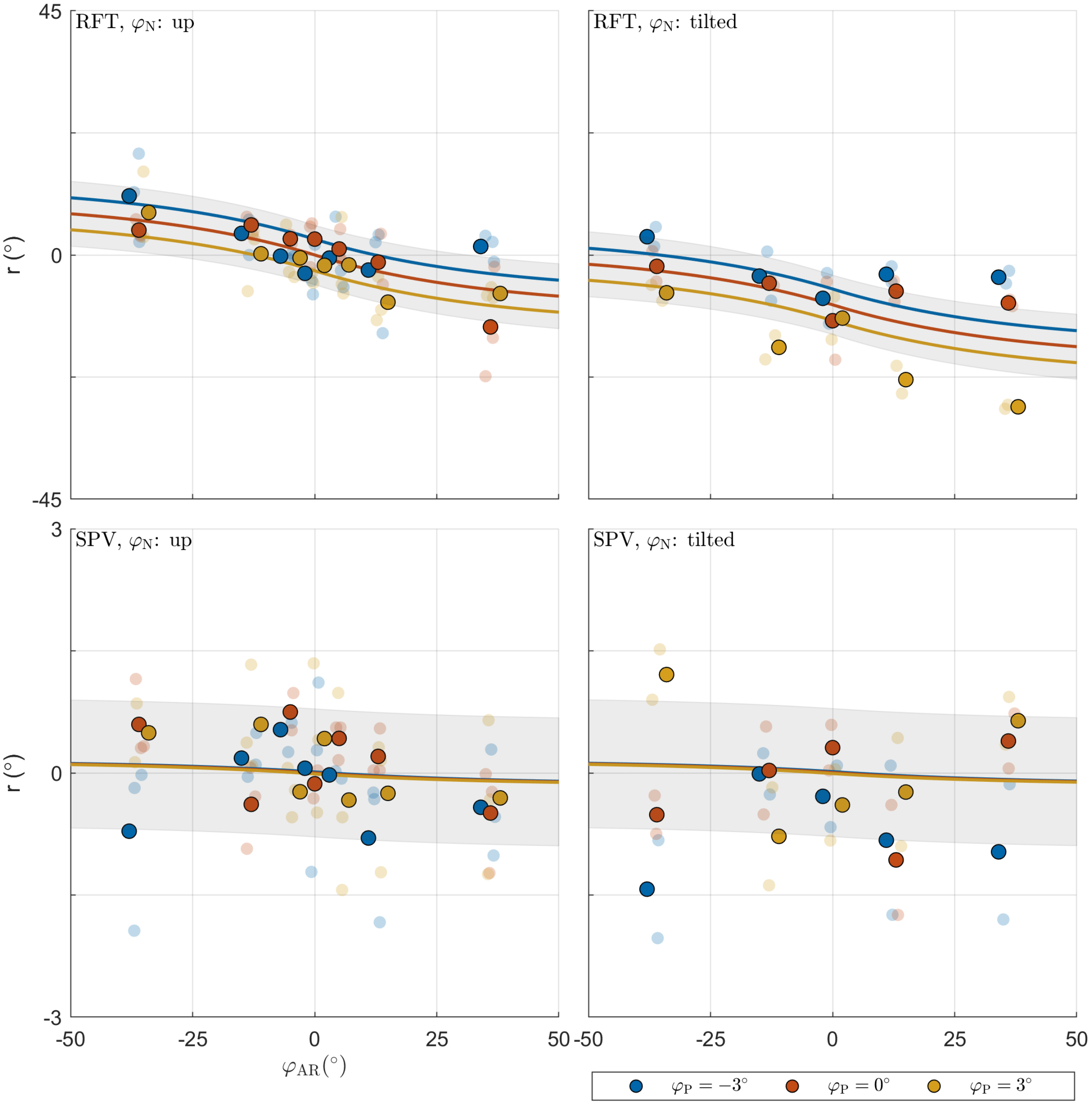

Participant id 10 (additional negative offset for neck tilt in SPV task; see main text)

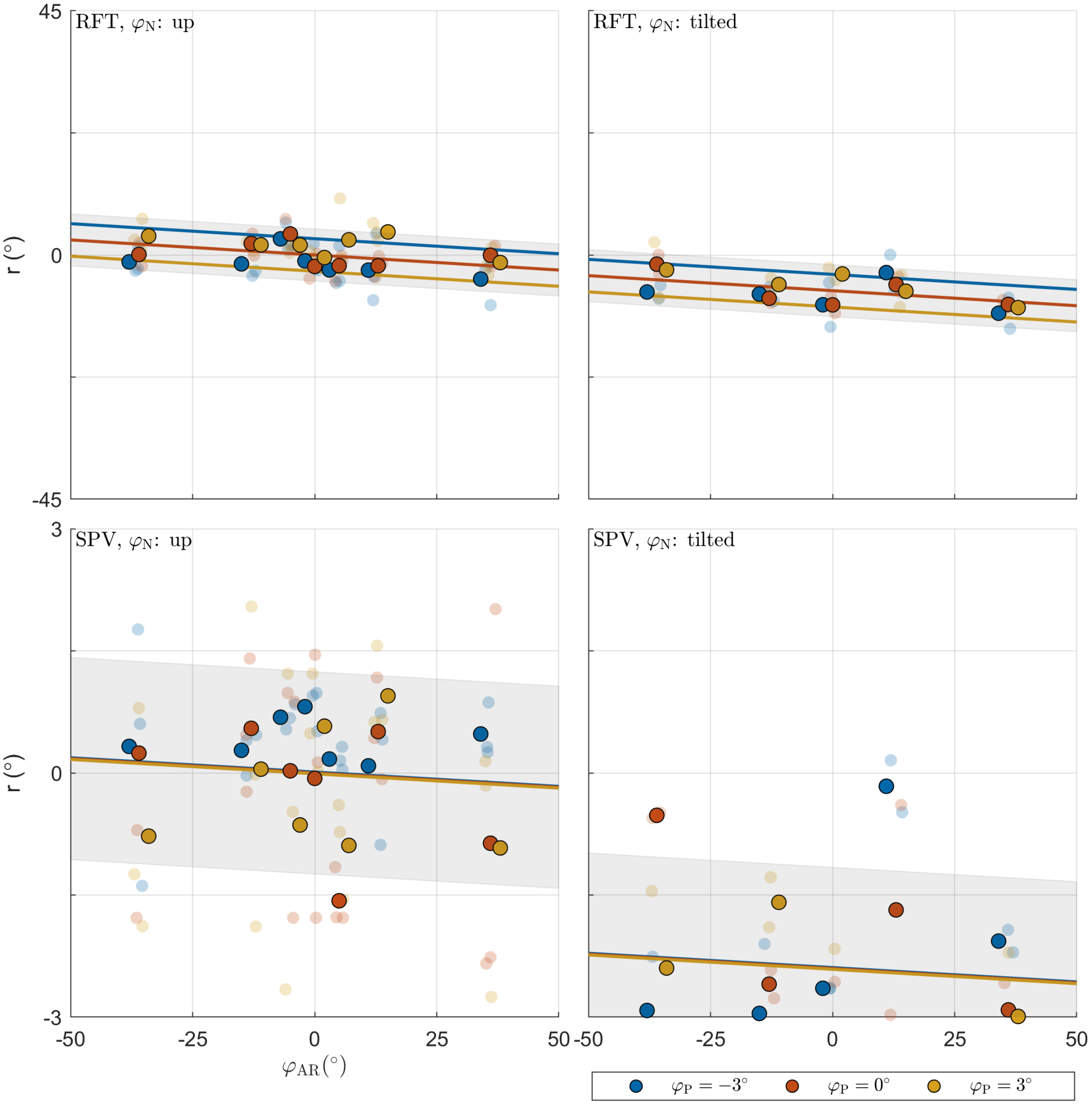

Supplement: Supplementary file 1 [file Presentation_1.pdf]
